# Bayesian credible subgroup identification for treatment effectiveness in time-to-event data

**DOI:** 10.1371/journal.pone.0229336

**Published:** 2020-02-26

**Authors:** Duy Ngo, Richard Baumgartner, Shahrul Mt-Isa, Dai Feng, Jie Chen, Patrick Schnell

**Affiliations:** 1 Merck & Co., Inc., Kenilworth, NJ, United States of America; 2 MSD Research Laboratories, MSD, London, United Kingdom; 3 Department of Statistics, Western Michigan University, Kalamazoo, Michigan, United States of America; 4 School of Public Health, Imperial College London, London, United Kingdom; 5 The Ohio State University College of Public Health, Columbus, Ohio, United States of America; Roswell Park Cancer Institute, UNITED STATES

## Abstract

Due to differential treatment responses of patients to pharmacotherapy, drug development and practice in medicine are concerned with personalized medicine, which includes identifying subgroups of population that exhibit differential treatment effect. For time–to–event data, available methods only focus on detecting and testing treatment–by–covariate interactions and may not consider multiplicity. In this work, we introduce the Bayesian credible subgroups approach for time–to–event endpoints. It provides two bounding subgroups for the true benefiting subgroup: one which is likely to be contained by the benefiting subgroup and one which is likely to contain the benefiting subgroup. A personalized treatment effect is estimated by two common measures of survival time: the hazard ratio and restricted mean survival time. We apply the method to identify benefiting subgroups in a case study of prostate carcinoma patients and a simulated large clinical dataset.

## 1 Introduction

A goal of clinical trials is to evaluate primary endpoints that describe comprehensive characteristics of the disease under study and allow for comparisons of treatments in an entire population. However, trial populations are often heterogeneous due to different demographics, medical history or genetic makeup among patients. In some cases, the efficacy of marketed treatments could not be replicated in follow–up clinical trials [1]. The inability to replicate study results in follow-up trials may be caused by different proportions of benefiting and non–benefiting subgroups of patients from experimental treatment compared to control. Recently, regulators and health technology assessment agencies worldwide have had a growing interest in identifying subgroups of patients who benefit from a treatment. Several methods to find such subgroups in clinical trials have been proposed in the literature [2–4].

Our study is motivated by a practical need for identifying subgroups of patients with improved time-to-event or survival outcomes. Many tree–based and model–based methods have been developed for time–to–event subgroup analysis [5–8]. Ballarini et al. [9] recently introduced a multiple regression model with a Lasso–type penalty to estimate benefiting subgroups based on estimates of the personalized treatment effect (PTE) and its post–selection confidence intervals. Traditionally, log–rank tests and Cox proportional hazard models have been used to compare treatment effects on an entire population. For example, researchers can identify subgroups with an overall *positive* treatment effect such as hazard ratio (HR) <1. However, this approach does not identify a benefiting subgroup in which *all* members defined by a set of observed baseline characteristics have a positive treatment effect. Likewise, the average treatment effect (ATE) is the average over the entire population of individual treatment effects, and it does not accurately represent each patient’s treatment effect.

Recently, the personalized treatment effects (PTEs) have been considered as a suitable alternative to the ATE for determining subpopulations of interest that benefit from a given treatment. Researchers have been focusing on estimating PTE at each predictive covariate point, that is, a set of baseline characteristics that predicts the patient’s response to a particular treatment. In a regression model, predictive covariates are incorporated in treatment–covariate interaction terms, and a hypothesis test of a null PTE is considered for each predictive covariate point. Two main issues with this approach are high multiplicity and low power to detect a treatment–covariate interaction [10–13]. In addition to these issues, Pocock et al. [3] points out that biological plausibility should be assessed along with consideration of the strength of evidence for heterogeneity in the treatment effect.

In this paper, we develop a Bayesian approach for subgroup analysis with time–to–event data based on recent advances in subgroup identification methodology proposed by Schnell et al. [14–16]. In a Bayesian framework, Schnell et al. [14] provide a two-step procedure to estimate a benefiting subgroup: (1) fit a regression model, and (2) construct bounding subgroups based on the posterior distribution of PTEs. Compared to previous methods, Schnell et al.’s method has several advantages, such as controlling for multiplicity and easily making statistical inferences from the full posterior distribution of the PTEs. This construction furnishes a pair of credible subgroups: one that is likely to be contained by the benefiting subgroup and one that is likely to contain the benefiting subgroup. The corresponding inferential statement is that every type of patient in one bounding subgroup benefits, and no type of patient outside the other subgroup benefits. These inferences are simultaneous [14] in contrast to non-simultaneous inferences available from tree-based methods. Here the simultaneous inferences mean that all covariate points corresponding to a specific subpopulation simultaneously have a treatment effect exceeding a specified threshold.

Inspired by the two–step procedure, our approach to identify benefiting subgroups for time–to–event endpoints is to define a Cox proportional hazard model and make statistical inferences from the full posterior distribution of the HR between two arms. In a randomized clinical trial with a time-to-event endpoint, HR is the common efficacy measure which represents the relative difference between two survival curves based on the proportional hazard (PH) assumption. When the PH assumption is violated, the HR does not appropriately represent the PTEs. An alternative approach is the restricted mean survival time (RMST) which is the area under the survival curve up to a particular time point. As shown in previous studies [17, 18], RMST is a robust and clinically interpretable measure of the survival time distribution without PH assumption. Moreover, Uno et al. [19, 20] advocate for using RMST to estimate treatment effects as an alternative to the HR. In our approach, we consider the difference in RMST (RMSTd) between two randomized arms at a certain follow–up time point as the PTEs.

Our methods are also tested on two time–to–event datasets: One from a trial in patients with prostate cancer, and another from a simulated Merck Sharp & Dohme, London, UK (MSD) clinical trial in patients with myocardial infarctions. The first dataset is publicly available [21] and has been analyzed in Ballarini et al.’s study [9]. Our findings are similar to those found by Ballarini et al. [9], but, in addition, our methods also identify the non–benefiting subgroups to the treatment. The second dataset is a simulated data based on a randomized and placebo-controlled study on the effect of vorapaxar in addition with aspirin for secondary prevention of thrombotic events. Scirica et al. [22] applied a Cox proportional hazards model for testing heterogeneous HRs across prespecified subgroups of interest. Our primary interest is searching for benefiting subgroups from vorapaxar treatment, without prespecification of subgroups for testing but only based on covariates which may have predictive value.

Our proposed method is an extension of Schnell et al. [14] to time–to–event endpoints. It is important for practical applications and widens significantly application area of this work. The most important property of our method is handling multiplicity, because it enables control the familywise Type I error rate and thus explicitly controls the probability of making any spurious claims of subgroup benefits. Our method is amenable to the confirmatory setting, rather than the methods that are focused on potential benefiting group discovery. For the discovery, the multiplicity issues are not critical as it would be addressed in subsequent confirmatory analyses. Therefore, these two approaches are complementary to each other.

The organization of the rest of the paper is as follows: In Section 2 we present the Bayesian credible subgroup method for time–to–event endpoint with Section 2.1 introducing the notation and PTEs, defining the log HR and the difference in RMST as PTEs in Section 2.2 and 2.3 respectively. A simulation study is provided in Section 3 which implements our Bayesian credible subgroup with these two difference PTEs. Finally, a detailed analysis of two clinical datasets are presented in Section 4 and 5, while our conclusions are discussed in Section 6. This paper also has accompanying supplementary material containing details simulation study on the performance of HR and difference in RMST.

## 2 Methods

For the purpose of identifying a benefiting subgroup, traditional approaches begin with a test for treatment–covariate interactions [3, 23]. These approaches have well–known limitations including low power due to the smaller sample size within subgroups and multiplicity adjustment for a larger number of subgroups under investigation. More importantly, results of interaction tests do not directly answer the question of which types of patients (covariates profiles) benefit from treatment. Rejecting the no interaction hypothesis detects treatment heterogeneity, but it does not provide you with the information of which covariate points correspond to positive conditional average treatment effect. To overcome these difficulties, in a Bayesian framework, we introduce the method of credible subgroups to simultaneously identify which types of patients benefit from treatment.

### 2.1 Notation and personalized treatment effect (PTE)

For an event time *T*, let *x* be a *p* × 1 vector of prognostic covariates, and *z* be a *q* × 1 vector of predictive covariates. Some covariates may appear in both *x* and *z*, and intercept terms may be included. Suppose that *T* is only partially observed during an experiment due to censoring, such as right censoring denoted by a random variable *C*. We assume that *T* is independent of *C* given *x*. Let *Y* = min(*T*, *C*) and *κ* be a failure indicator, i.e. *κ* = 1 for *T* ≤ *C* and 0 otherwise (See S1 File Moreover, we consider the time–to–event data to consist of *n* subjects who were randomly assigned to one of two treatments, i.e. *θ* = {0, 1}, and only predictive covariates *z* interact with treatment indicator *θ* in our model fit. In this scenario, the observed data consist of *n* independent realizations of {(*Y*_*i*_, *x*_*i*_, *z*_*i*_, *κ*_*i*_, *θ*_*i*_)} for *i* = 1, …, *n*.

For a two–arm study with censoring, a common non–parametric approach to compare the survival distributions between two treatment groups is the log rank test. This test is based on a series of 2 × 2 contingency tables constructed at each observed failure time. A semi–parametric approach using the Cox regression model is commonly applied to investigate the effect of covariates on the HR. These two approaches measure the average treatment effect on the entire study population, which cannot be used to identify which patient benefits from a treatment. Personalized treatment effects (PTEs) are becoming widely used to determine subgroups of patients who most benefit from a treatment. We denote Δ as a PTE for a subject with covariate vectors *x*_*i*_ and *z*_*i*_, and precisely define Δ for the log HR and RMST differences as measures for PTE in the following sections.

### 2.2 The log HR as a PTE

The Cox model, which is commonly used for the analysis of time–to–event data, has the following form:

$$\begin{array}{c} \lambda \left(t|x_{i},z_{i},\theta_{i}\right)=\lambda_{0}\left(t\right)\exp\left(x_{i}^{\prime }\beta +\theta_{i}z_{i}^{\prime }\gamma \right), \end{array}$$ (1)

where λ(*t*|*x*_*i*_, *z*_*i*_, *θ*_*i*_) is the hazard function at time *t* for the *i*th subject with covariates *x*_*i*_ and *z*_*i*_, λ_0_(*t*) is the unspecified baseline hazard function, and *β* and *γ* are *p* × 1 and *q* × 1 vectors of regression coefficients, respectively. Here *x*′ denotes the transpose of vector *x*. We include the interaction terms between *z* and *θ* in the model, and the PTE for a patient with covariate *z* is

$$\begin{array}{c} \Delta_{H}\left(z_{i}\right)=\frac{\lambda (t|x_{i},z_{i},\theta_{i}=1)}{\lambda (t|x_{i},z_{i},\theta_{i}=0)}=\exp\left(z_{i}^{\prime }\gamma \right), \end{array}$$ (2)

which is a ratio between the hazards of a patient with treatment *θ* = 1 and *θ* = 0.

If a given element of *γ* is positive, then higher values of the corresponding element of *z* would indicate that the subject has a higher hazard for treatment *θ*_*i*_ = 1 or shorter survival than the subject with treatment *θ*_*i*_ = 0. Therefore, to determine the characteristics of subjects who benefit from treatment *θ*_*i*_ = 1, we set a predetermined threshold of clinical significance 0 < *δ*_*H*_ ≤ 1, and search for the points *z*_*i*_ such that

$$\begin{array}{c} \Delta_{H}\left(z_{i}\right)=\exp\left(z_{i}^{\prime }\gamma \right)<\delta_{H}. \end{array}$$ (3)

Alternatively,

$$\begin{array}{c} \log\Delta_{H}\left(z_{i}\right)=z_{i}^{\prime }\gamma <\log\delta_{H}, \end{array}$$ (4)

where logΔ_*H*_(*z*_*i*_) is the log HR evaluated at points *z*_*i*_. In subgroup analysis for time-to-event data, we want to find a subgroup in which *every* covariate point for a subject has a conditional log HR less than log *δ*_*H*_. This approach is distinguished from finding a subgroup whose overall log HR is less than log *δ*_*H*_ in the sense that such subgroup can contain members with higher log HR than log *δ*_*H*_.

From Eq (2), the HR from the Cox regression model between two subjects is constant over time. This assumption often fails in time-to-event data. For example, non-proportional hazard is present in immuno-oncology trials due to delayed treatment effect and/or functional cure. When the proportional hazard assumption does not hold, the Δ_*H*_(*z*_*i*_) may no longer provide suitable PTE for a subject. An alternative approach is to use the RMST which is a robust measure of the survival time distribution without relying on the proportional hazard (PH) assumption. We present the RMST in the next section and describe how it may be used to define the PTE for a subject.

### 2.3 Difference in restricted mean survival times (RMSTd) as a measure of PTE

The RMST *ψ* of a random variable failure time *T* is the mean of the time–to–event *ζ* = min(*T*, *ν*) limited to some cutoff time point *ν* > 0. In other words, the RMST is the area under the survival curve *S*(*t*) between *t* = 0 to *t* = *ν* and can be expressed as

$$\begin{array}{c} \psi =E\left(\zeta \right)=\int_{0}^{\nu }S\left(t\right)\;dt, \end{array}$$ (5)

In a randomized two–arm clinical trial, let *S*(*t*|*θ* = 1) and *S*(*t*|*θ* = 0) be the survival functions for the treatment *θ* = 1 and *θ* = 0 respectively. The RMSTd between two arms from *t* = 0 to *t* = *ν* is defined as

$$\begin{array}{c} \Delta_{Rd}=\psi_{\theta =1}-\psi_{\theta =0}=\int_{0}^{\nu }[S(t|\theta =1)-S(t|\theta =0)]\;dt, \end{array}$$ (6)

which is the difference in area between the two survival curves. Alternatively, one can also define the ratio of RMST between two arms such as $\Delta_{Rr}=\frac{\int_{0}^{\nu }S\left(t|\theta =1\right)\;dt}{\int_{0}^{\nu }S\left(t|\theta =0\right)\;dt}$.

In this paper, we use the Δ_*Rd*_ as the PTE for a subject, and estimate the two survival functions *S*(*t*|*θ* = 0) and *S*(*t*|*θ* = 1) in Eq 6 on the grid of subgroup–defining covariates in order to compute the Δ_*Rd*_. A common approach is to obtain a Kaplan–Meier estimator, but that would not take the covariates into account. Thus we employ the conventional Cox proportional hazard model to estimate these two survival functions. Note that we could still employ fixes to PH violations (e.g., time-dependent covariates or effects) without having to worry about reporting time-dependent hazard ratios.

Moreover, if *T* is years to death and *S*(*t*|*θ* = 1)>*S*(*t*|*θ* = 0) for *t* ∈ [0, *ν*], the interpretation of Δ_*Rd*_ is that a subject has the *ν*-year life expectancy higher in treatment *θ* = 1 than *θ* = 0, so this subject could be benefiting from treatment *θ* = 1. Similar to the HR, to identify benefiting subjects from treatment *θ* = 1, we set Δ_*Rd*_ to be greater than some predetermined threshold of clinical significance *δ*_*R*_ > 0. Compared to Δ_*H*_, the advantage of Δ_*Rd*_ would not rely on the PH assumption. However, if it were to hold, possible questions of interest would be “is there relationship between the two PTE measurements?” and “if the investigators knows *δ*_*H*_, what is the corresponding *δ*_*R*_ (or vice versa)?” To address these questions, we show in S1 File that in parametric settings and when *δ*_*H*_ = 1 and *δ*_*R*_ = 0, Δ_*H*_ and Δ_*Rd*_ are the same for determining whether a patient benefits from the treatment. Thus we use these predetermined significance values in our simulation study (Section 3). In the following section, we show how these PTE measurements are related to subgroup identification problem in the Bayesian framework.

### 2.4 Bayesian credible subgroups for time-to-event data

There are numerous non-parametric and parametric methods for PTEs to identify who benefits from a treatment given their baseline characteristics. Among them, Berger et al. [23] proposed a Bayesian model selection using tree–based priors that provide the posterior distribution for use in statistical inference. Recently, Ballarini et al. [9] developed the predicted individual treatment effect (PITE) using a multiple regression framework with a Lasso–type penalty for model selection, and provided confidence intervals for the PTEs. Subgroup identification will be evaluated based on these confidence intervals. While the inferences from tree–based methods are not simultaneous, the PITE methods does not address the multiplicity issue. To overcome these limitations, Schnell et al. [14] proposed a Bayesian credible subgroups methods for continuous endpoints which addressed both simultaneous inference and multiplicity. In this paper, we extend their approach to survival endpoints by using two summaries commonly used in clinical trials: the log HR and RMSTd. In the following sections, we first present the Bayesian credible subgroups methods, and introduce a Bayesian approach to obtain the inference for the time–to–event PTEs. Then we construct simultaneous credible bands from those inferences.

#### 2.4.1 Bayesian credible subgroups

A goal of the Bayesian credible subgroups method [14] is to estimate a set of subject baseline covariate points for which a subject would benefit from the treatment according to a PTE. More precisely, let Z be a covariate space, this approach searches for the set of covariate points $B_{H}=\left\{z\in Z:\Delta_{H}\left(z\right)<\log\left(\delta_{H}\right)\right\}$ when the PTE is measured as the log HR, or $B_{Rd}=\left\{z\in Z:\Delta_{Rd}\left(z\right)>\delta_{R}\right\}$ for the RMSTd. In a Bayesian framework, a common estimator for *B* includes the points z∈Z whose posterior probability of having the log HR less than log(*δ*_*H*_) (or greater than *δ*_*R*_ for Δ_*Rd*_) given observed data is greater than (1 − *α*), where 1 − *α* is a credible level, and can be expressed as

$$\begin{array}{c} {\hat{B}}_{H,\alpha }=\left\{z\in Z:P(\Delta_{H}\left(z\right)<\delta_{H}\;|\;\text{Data})>1-\alpha \right\} \end{array}$$ (7)

for the PTE of log HR or

$$\begin{array}{c} {\hat{B}}_{Rd,\alpha }=\left\{z\in Z:P(\Delta_{Rd}\left(z\right)>\delta_{R}\;|\;\text{Data})>1-\alpha \right\} \end{array}$$ (8)

for the PTE of RMSTd. To control for multiplicity, the credible subgroup pair (*D*, *S*) consists of an exclusive credible subgroup *D* and inclusive credible subgroup *S* such that *P*(*D* ⊆ *B* ⊆ *S*|Data)>1 − *α*. This means that with posterior probability (1 − *α*), *D* contains only covariate points *z* for which the types of subjects benefit from the treatment, while *S* includes all types of subjects who benefit.

**Fig 1:**
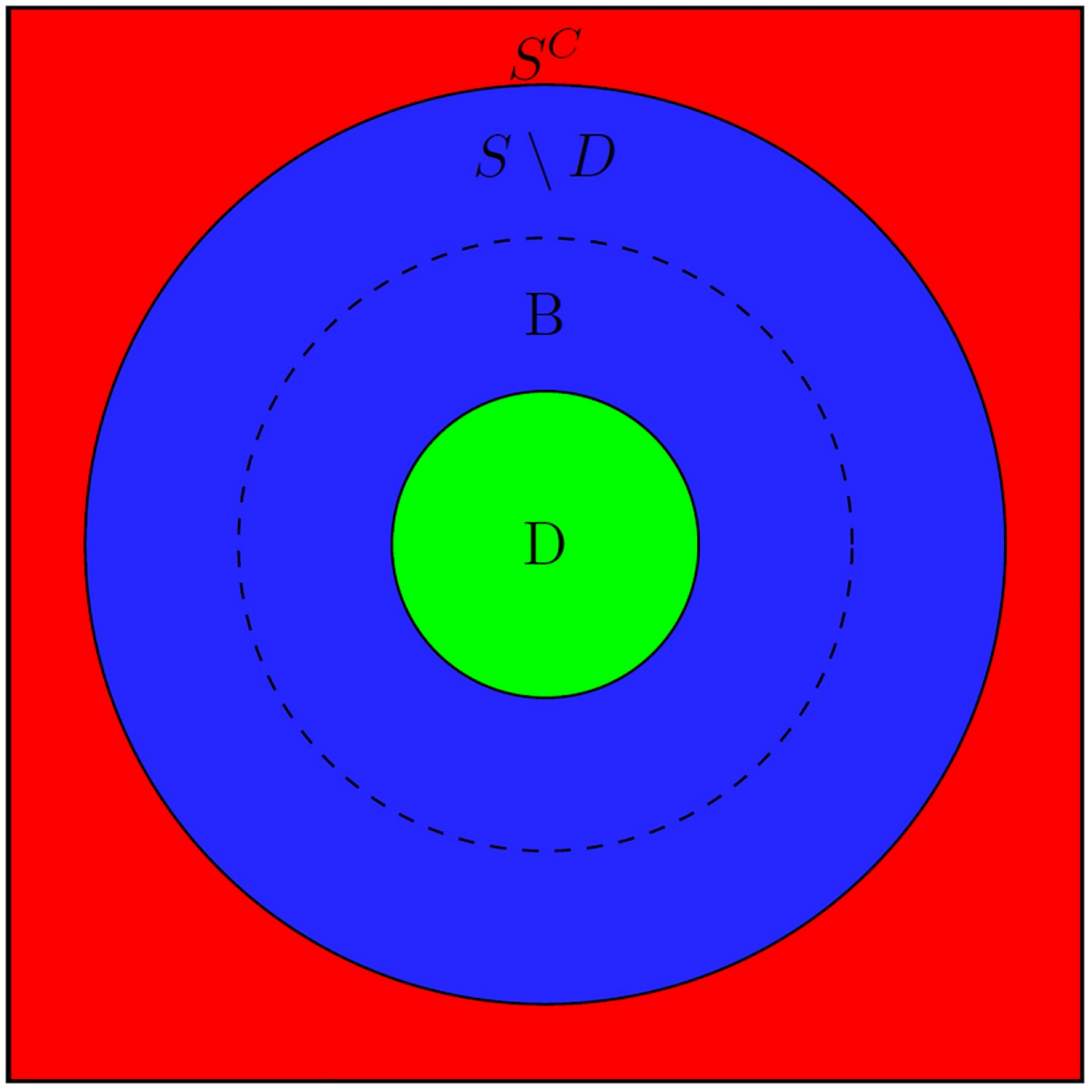
Illustration of credible subgroups. B contains true type of patients who benefit (enclosed by dashed line) while D includes only type of patients who benefit (green). Moreover, type of patients in *S*\*D* require more information (blue), and those in *S*^*C*^ have no benefit (red).

Fig 1 illustrates the covariate space Z divided into three regions. The region *B* enclosed by dashed circle represents the true benefiting subgroup which we wish to estimate by a pair (*D*, *S*). Here the green region *D* includes the types of patients who have evidence of benefit whereas patients in the red region *S*^*C*^ have no benefit. Finally, the blue region is a region of uncertainty that needs more information.

The Bayesian credible subgroups method described above is a two-step procedure: (1) define a model, fit a regression and obtain the marginal posterior of the PTE onto the given covariates; and (2) compute the bounds and obtain a pair (*D*, *S*). In the first step, we fit a Cox regression model in a Bayesian framework to get the marginal posterior of coefficients of predictive covariates that interact with treatment choice (in Section 2.4.2). In the second step, we describe the method to compute the credible subgroups (in Section 2.4.3).

#### 2.4.2 Bayesian estimation in time–to–event analysis

Several methods have been proposed for Bayesian analysis of the Cox proportional hazards model with right censored data [24–26]. In this section, we review the commonly used gamma process prior for the Cox regression model. In Eq 1, we need to specify priors on ***β*** = (*β*′, *γ*′)′ and the baseline hazard function λ_0_(*t*). A typical prior for *β* is a *N*_*p*+*q*_(*μ*_0_, Σ_0_) which is a *p* + *q*-variate normal distribution with mean vector *μ*_0_ and covariate Σ_0_. We choose a nonparametric gamma process prior on the cumulative baseline hazard. This prior partitions the observed survival time into intervals, such as 0 < *s*_1_ < *s*_2_ < … < *s*_*J*_ where *s*_*J*_ > *y*_*i*_ for *i* = 1, …, *n*. Thus, we have *J* disjoint intervals. The observed data $D=\{x,z,R_{j},M_{j}\;\text{for}\;j=1,\ldots ,J\}$ will be grouped within these intervals where *R*_*j*_ and *M*_*j*_ are the risk set and failure set of the *j*th interval (*s*_*j*−1_, *s*_*j*_], respectively. Let *h*_*j*_ = *H*_0_(*s*_*j*_) − *H*_0_(*s*_*j*−1_) be the increment in the cumulative baseline hazard $H_{0}\left(t\right)=\int_{0}^{t}\lambda_{0}\left(k\right)\;dk$ in the *j*th interval. Thus, the *h*_*j*_’s are independent increments in disjoint intervals and

$$\begin{array}{c} h_{j}∼G\left(\alpha_{j}-\alpha_{j-1},b\right), \end{array}$$ (9)

where G(α,b) denote the gamma distribution with shape parameter *α* > 0 and scale parameter *b* > 0, such that *α*_*j*_ = *bH*(*s*_*j*_) with an increasing function *H*(*s*_*j*_). *H* and *b* are hyperparameter for *h*_*j*_, and *H*_*b*_ is a specified parametric cumulative hazard function evaluable at the endpoints of the time intervals, and the scalar *b* is a weight about the mean. Therefore, the observed likelihood function is

$$\begin{array}{c} L(\beta ,h\;|\;D)\propto \prod_{j=1}^{J}l_{j}, \end{array}$$ (10)

where $l_{j}=\exp[-h_{j}\sum_{k\in R_{j}\backslash M_{j}}\exp\left(x_{k}^{\prime }\beta \right)]\prod_{m\in M_{j}}[1-\exp(-h_{j}\exp(x_{m}^{\prime }\beta ))]$, and **x** is a combined vector of ${\left(x_{i}^{\prime },\theta z_{i}^{\prime }\right)}^{\prime }$.

From the prior distribution for ***β*** and **h** = (*h*_1_, …, *h*_*J*_)′ described above, the joint posterior of ***β*** and **h** is

$$\begin{array}{c} P(\beta ,h\;|\;D)\propto \prod_{j=1}^{J}[l_{j}h_{j}^{\alpha_{j}-\alpha_{j-1}-1}\exp\left(-bh_{j}\right)]\exp(\frac{-1}{2}\left(\beta -\mu_{0}\right)\Sigma_{0}^{-1}\left(\beta -\mu_{0}\right)). \end{array}$$ (11)

From Eq 11, the conditional distribution of *β*_*i*_ given **h**, and ***β***^(−*i*)^ denoted the ***β*** vector without the *i*th component is

$$\begin{array}{c} P(\beta_{i}\;|\;\beta^{\left(-i\right)},h,D)\propto \prod_{j=1}^{J}l_{j}\exp(\frac{-1}{2}\left(\beta -\mu_{0}\right)\Sigma_{0}^{-1}\left(\beta -\mu_{0}\right)). \end{array}$$ (12)

where *i* = 1, …, *p* + *q*. Similarly, the conditional distribution of *h*_*j*_ is

$$\begin{array}{c} P(h_{j}\;|\;h^{\left(-j\right)},\beta ,D)\propto h_{j}^{\alpha_{j}-\alpha_{j-1}-1}\exp[-h_{j}(\sum_{k\in R_{j}\backslash M_{j}}\exp\left(x_{k}^{\prime }\beta \right)+b)]. \end{array}$$ (13)

The posterior distribution of the parameter of interest can be obtained from these full conditional distributions in Eqs 12 and 13 by Gibbs sampling in a straightforward way. Based on the posterior of coefficients of predictive covariates, we can obtain the posterior of Δ_*H*_(*z*). For Δ_*Rd*_(*x*, *z*), we first compute the posterior distribution for survival function *S*(*t*) = exp(−*H*_0_(*t*)exp(**x**′ *β*)) for each treatment and then obtain the difference between two treatments (See S1 File for more details of constructing the posterior of Δ_*H*_(*Z*) and Δ_*Rd*_(*x, Z*)). We implement our proposed method from an R package **spBayesSurv** provided by Zhou et.al. [27]. For ease of notation, we denote Δ(*z*) for a general PTE and proceed to construct credible subgroups in the next section. **Remark**: We presented Bayesian estimation of the Cox regression model by using the gamma process prior. However, there are several advanced Markov chain Monte Carlo sampling techniques that were proposed that could be used in this context. They include slice sampling [28] and Hamiltonian Monte Carlo [29]. There are also associated software packages available, e.g. Stan [30], or the R packages such as MfUSampler [31], and sns [32] which provide a wider range of model specifications and which can be used for Bayesian survival analysis. A notable example represents the R package BSGW [33].

#### 2.4.3 Credible subgroups estimation

A goal of constructing credible subgroups based on the posterior of Δ(*z*) is to control the multiplicity in testing Δ(*z*) at every covariate point and to provide two credible subgroups (*D*, *S*) which bound the benefiting subgroup *B*. These simultaneous credible bands over the covariate space Z can be constructed as

$$\begin{array}{c} \Delta \left(z\right)\in \hat{\Delta }\left(z\right)\pm \sqrt{W_{\alpha }\text{Var}\left(\Delta \left(z\right)\right)}, \end{array}$$ (14)

where *W*_*α*_ is the 1 − *α* quantile of the distribution of $W=\underset{z\in Z}{\sup}\frac{{\left(\Delta \left(z\right)-\hat{\Delta }\left(z\right)\right)}^{2}}{\text{Var}(\Delta (z))}$ and $\hat{\Delta }\left(z\right)$ is the posterior mean of Δ(*z*). Therefore, in a case of Δ(*z*) ≡ Δ_*H*_(*z*), the exclusive credible subgroup *D* is given by

$$\begin{array}{c} D=\{z\in Z:{\hat{\Delta }}_{H}\left(z\right)+\sqrt{W_{\alpha }\text{Var}\left(\Delta \left(z\right)\right)}<\delta_{H}\} \end{array}$$ (15)

and inclusive credible subgroup *S* is

$$\begin{array}{c} S=\{z\in Z:{\hat{\Delta }}_{H}\left(z\right)-\sqrt{W_{\alpha }\text{Var}\left(\Delta \left(z\right)\right)}\leq \delta_{H}\}. \end{array}$$ (16)

Note that Δ(*z*) was sampled from the exact posterior as described in previous section, and then we used Gaussian approximation to obtain the simultaneous credible bands in Eqs 15 and 16. In this paper, we construct the asymptotic credible bands since the posterior distributions for representative covariate points in our applications were approximately Gaussian (See S1 File). Moreover, Schnell et al. [15] proposed a quantile–based simultaneous credible band when Δ(*z*) is non–Gaussian.

## 3 Simulation study

In this section, we conduct extensive simulation studies to evaluate the performance of Bayesian credible subgroups in simulated time–to–event data under the PH and non PH assumptions.

### 3.1 Simulation study under proportional hazard assumption

For the Cox proportional hazard model, we assume that the hazard function for the *i*^*th*^ individual (*i* = 1, …, *n*) is

$$\begin{array}{c} \lambda_{i}\left(t\right)=\lambda_{0}\left(t\right)\exp\left(x_{i}^{\prime }\beta +\theta_{i}z_{i}^{\prime }\gamma \right), \end{array}$$ (17)

where *x*_*i*_ = (*x*_*i*1_, *x*_*i*2_)′ and *z*_*i*_ = (1, *z*_*i*1_, *z*_*i*2_)′ are the vectors of prognostic and predictive covariates respectively. Then *β* = (*β*_1_, *β*_2_)′ and *γ* = (*γ*_1_, *γ*_2_, *γ*_3_)′ are vector of coefficients of *x*_*i*_ and *z*_*i*_, respectively. Moreover, we assume a Weibull baseline hazard, i.e. λ_0_(*t*) = λ*ν*(λ*t*)^*ν*−1^ where λ and *ν* are the scale and shape parameters respectively. Then the inverse of the cumulative hazard function is $H_{0}^{-1}\left(t\right)={\left(\lambda^{-1}t\right)}^{1/\nu }$. If *U* is uniformly distributed on [0, 1], the survival time *T*_*i*_ can be generated as

$$\begin{array}{c} T_{i}=H_{0}^{-1}[-\frac{\log(U)}{\exp(x_{i}^{\prime }\beta +\theta_{i}z_{i}^{\prime }\gamma )}]=-{\left[\frac{\log(U)}{\lambda \exp(x_{i}^{\prime }\beta +\theta_{i}z_{i}^{\prime }\gamma )}\right]}^{1/\nu }. \end{array}$$ (18)

Suppose that *C*_*i*_ are the censoring times, drawn from an exponential distribution Exp(*a*). Due to censoring, we observe *Y*_*i*_ = min(*T*_*i*_, *C*_*i*_) and censoring indicators *κ*_*i*_. For parameters of the simulation time–to–event data, we set λ = 0.05 and *ν* = 1.1 for a Weibull baseline hazard and a rate *a* = 0.02 for the censoring time. Furthermore, we let *x*_*i*1_ = *z*_*i*1_ = {0, 1} with equal probability, and *x*_*i*2_ = *z*_*i*2_ be uniformly distributed on the interval (−3, 3), and we only consider two arms, i.e. *θ*_*i*_ = {0, 1}.

Then we perform diagnostic test for credible subgroups with different sample sizes (*n* = 50, 100, 500, 1000) and at different credible levels (0.4, 0.6, 0.8, 0.95). Finally, we consider three cases of *β* with different values of *γ*:

1. The prognostic features have no effect *β* = (0, 0), and we set *γ* = (0, 0, 0), (0.1, 0.1, 0.1), (1, 1, 1), (1, −1, 3),
2. The prognostic features have moderate effect *β* = (0.2, 0.2), and we set *γ* = (1, 1, 1),
3. The prognostic features have higher effect *β* = (1, −2), and we set *γ* = (1, 0.1, 1).

Following the same criteria in Schnell et al.’s simulation study [14], we report five metrics: (1) total coverage measures the frequency with which *D* ⊆ *B* ⊆ *S* for some fixed value *z*; (2) the pair size measures the proportion of covariate points in the uncertainty region *S*\*D*; (3) specificity and sensitivity of *D* measures how well the credible subgroup *D* aligns with benefiting subgroup *B*; and (4) mean squared error (MSE) of the treatment effects compares the estimated treatment effects to the true values.

As shown in S1 File, the PTEs measured by the log HR and RMSTd yield the same result when *δ*_*H*_ = 1 and *δ*_*R*_ = 0. We provide three simulation studies as follows:

- Simulation 1: run using the same simulation time–to–event data for log HR and RMSTd when *δ*_*H*_ = 1 and *δ*_*R*_ = 0.
- Simulation 2: run only for log HR at different thresholds *δ*_*H*_ = {0.2, 0.5, 1, 2}.
- Simulation 3: only for the RMSTd at *δ*_*R*_ = {−1, 0, 1}.

For each simulation study, we simulate 1000 data sets, and for each data set, we use 1000 posterior draws kept after 500 burn-in iterations. Table 1 reports the average summary statistics for Simulation 1 at an 80% credible level. When the effect sizes are relatively small, the benefiting subgroup is empty, and sensitivity of *D*, which is the proportion of the benefiting subgroup *B* included in the exclusive credible subgroup *D*, is not calculable. This is represented with ‘NaN’ in the table. Overall, we find that the total coverage is always greater than 80% for both PTE approaches. When the sample size and/or effective size are increasing, the credible size is decreasing except when *β*’s are zero. The RMSTd approach has larger credible size than log HR for *n* = 50 and 100 but smaller or similar credible size for larger *n*. Moreover, both PTE approaches have similar specificity of *D*, which is the proportion of the non–benefiting subgroup *B* not included in the exclusive credible subgroup *D*. Compared to the RMSTd approach, the log HR tends to have higher sensitivity of *D* for a small sample size (*n* = 50) but low sensitivity for a large sample size (*n* = 1000). Finally, the RMSTd approach has small MSE in most scenarios. In general, both approaches show similar trends. Simulation results for other simulation settings, and comparison between the proposed Bayesian credible subgroup with the pointwise method, i.e. without multiplicity correction [14], are also reported in S1 File. We found that moving from pointwise method to our proposed methods, there is increasing in credible pair size, specificity of D, but smaller sensitivity of D.

**Table 1:** Simulation 1 results: Average summary statistics for 80% credible level.

Sample size	Truth	Total Coverage		Credible Pair Size		Sensitivity of D		Specificity of D		MSE	
		log HR	RMSTd	log HR	RMSTd	log HR	RMSTd	log HR	RMSTd	log HR	RMSTd
50	(0,0,0,0,0)	0.88	0.88	0.94	0.93	NaN	NaN	0.97	0.97	0.44	0.48
	(0,0,0.1,0.1,0.1)	0.9	0.86	0.94	0.93	0.04	0.04	0.99	0.98	0.46	0.51
	(0,0,1,1,1)	0.92	0.94	0.32	0.38	0.45	0.47	0.99	0.99	0.46	0.38
	(0,0,1,-1,3)	0.92	0.94	0.12	0.19	0.97	0.97	0.99	0.99	0.96	0.28
	(0.2,0.2,1,1,1)	0.9	0.92	0.31	0.45	0.45	0.44	0.99	0.99	0.43	0.36
	(1,-2,1,0.1,1)	0.96	0.97	0.35	0.55	0.52	0.12	1	1	0.59	0.26
100	(0,0,0,0,0)	0.9	0.89	0.95	0.95	NaN	NaN	0.98	0.98	0.18	0.25
	(0,0,0.1,0.1,0.1)	0.89	0.91	0.9	0.91	0.08	0.08	0.99	0.99	0.2	0.26
	(0,0,1,1,1)	0.92	0.92	0.21	0.21	0.72	0.73	0.99	0.99	0.22	0.22
	(0,0,1,-1,3)	0.91	0.92	0.08	0.08	1	1	0.99	0.99	0.49	0.21
	(0.2,0.2,1,1,1)	0.89	0.9	0.21	0.22	0.72	0.71	0.99	0.99	0.23	0.21
	(1,-2,1,0.1,1)	0.96	0.96	0.21	0.3	0.79	0.57	1	1	0.28	0.2
500	(0,0,0,0,0)	0.88	0.9	0.95	0.95	NaN	NaN	0.97	0.98	0.03	0.05
	(0,0,0.1,0.1,0.1)	0.94	0.92	0.77	0.77	0.08	0.08	1	1	0.03	0.05
	(0,0,1,1,1)	0.9	0.92	0.13	0.13	1	1	0.99	0.99	0.08	0.06
	(0,0,1,-1,3)	0.88	0.86	0.06	0.05	1	1	0.98	0.98	0.31	0.05
	(0.2,0.2,1,1,1)	0.94	0.92	0.13	0.13	1	1	0.99	0.99	0.08	0.06
	(1,-2,1,0.1,1)	0.92	0.92	0.12	0.12	1	1	0.99	0.99	0.07	0.07
1000	(0,0,0,0,0)	0.9	0.89	0.97	0.96	NaN	NaN	0.98	0.98	0.01	0.03
	(0,0,0.1,0.1,0.1)	0.94	0.94	0.59	0.58	0.15	0.18	1	0.99	0.02	0.03
	(0,0,1,1,1)	0.88	0.88	0.13	0.13	1	1	0.99	0.99	0.06	0.03
	(0,0,1,-1,3)	0.87	0.88	0.06	0.06	1	1	0.98	0.98	0.28	0.03
	(0.2,0.2,1,1,1)	0.86	0.89	0.12	0.13	1	1	0.98	0.99	0.07	0.04
	(1,-2,1,0.1,1)	0.93	0.93	0.11	0.11	1	1	0.99	0.99	0.05	0.04

Multiplicity is incorporated into the simulation framework as the “total coverage” metric. Total coverage is the rate at which the true benefiting subgroup is both contained in the inclusive credible subgroup and contains the exclusive credible subgroup. A coverage failure corresponds to a family–wise error. The credible subgroup method should have a total coverage rate equal to the credible level, whereas a method not accounting for multiplicity would have lower total coverage due to that multiplicity.

### 3.2 Simulation study under nonproportional hazard assumption

When the PH assumption is violated, the HR may not accurately represent PTEs, so RMST summaries are used to estimate PTEs as an alternative approach to the HR. The simulation study in this section aims to investigate the performance of RMSTd for subgroup identification in a case of the nonproportional hazard.

From Eq 17, we simulated two groups with different hazard rates. The treatment group, i.e. *θ*_*i*_ = 1, had a constant exponential hazard with rate λ_0_(*t*) = 0.01. The control group, i.e. *θ*_*i*_ = 0, had a piecewise exponential hazard with rate

$$\begin{array}{c} \lambda_{0}\left(t\right)=\{\begin{array}{cc} 0.01 & 0\leq t<t_{c}\\ 0.1 & t_{c}\leq t. \end{array} \end{array}$$ (19)

Under this nonproportional hazard model, the hazard ratio between two treatments for subject *i* is $\exp(z_{i}^{\prime }\gamma )$ until time *t*_*c*_ and then there is an abrupt change to a rate of $\exp(z_{i}^{\prime }\gamma )/10$.

The first step of determining the two bounded subgroup pairs is to obtain the joint posterior sample of the PTEs at each covariate points. For the RMSTd, we employ a fully nonparametric Bayesian accelerated failure time (AFT) model proposed by Henderson et. al. [34]. It directly models the log-failure time as a sum of a regression function of covariates and residual. The conditional mean function is modeled using Bayesian additive regression trees (BART). The residual is modeled using a location-mixture of Gaussian distributions with a centered Dirichlet process as prior. We compute the survival functions of the non–parametric AFT model for each treatment, then take the difference between these survival functions to obtain the RMSTd at each covariate point.

The settings for the prognostic covariates *x*, the predictive covariates *z*, treatment indicator *θ* and censoring rate are similar to settings in simulation study under PH assumption. We considered the true values of coefficients *β* = (0.7, 0.7) and *γ* = (0.5, −0.5, −0.5). Then we simulated 1000 data sets, and for each data set, we used 1000 posterior draws kept after 500 burn-in iteration. Finally, we chose *δ*_*Rd*_ = 0 and *t*_*c*_ = 30. The RMSTd were computed at the change point *t*_*c*_ up to *t*_*c*_ + 50 when the treatment effect shows up during this time interval. The results for 80% credible subgroup pairs are presented in Table 2, and S1 File provides results for other credible levels.

**Table 2:** Average summary statistics for 80% credible subgroup pairs under nonproportional hazard assumption.

Sample Size	Total Coverage RMSTd	Credible Pair Size RMSTd	Sensitivity of D RMSTd	Specificity of D RMSTd	MSE RMSTd
50	0.76	0.5	0.52	0.76	0.68
100	0.79	0.36	0.68	0.79	0.58
500	0.86	0.17	0.88	0.86	0.43
1, 000	0.92	0.15	0.91	0.92	0.38

When the sample size increases, the credible pair size is smaller, and there is greater total coverage and improved sensitivity and specificity of D. Moreover, total coverage is above 80% for large sample size (*n* = 500, 1000) and close to 80% for small and moderate sample size (*n* = 50, 100). Table 2 also shows that a RMSTd approach tends to well estimate PTEs with respect to effect MSE. The results of this simulation suggest the RMSTd approach is appropriate to identify benefiting subgroups in a case of nonproportional hazards.

## 4 Analysis of the prostate cancer dataset

A prostate cancer dataset is publicly available [21] and has been analyzed in Rosenkranz et al. [35] for exploratory subgroup analysis by model selection. Ballarini et al. [9] have proposed the predicted individual treatment effect (PITE) to identify subgroups of patients who benefit from treatment. In this section, we illustrate the Bayesian credible subgroups method using the prostate cancer dataset, and compare our results with published results using PITE [9].

We include the 475 patients with complete data in our analysis as in the previously published studies. Each subject was randomly assigned either to a combination of placebo and the lowest dose level of diethyl stilbestrol (control group) or higher doses (treatment group). Moreover, we included the same covariates and interaction terms as used in Ballarini et al. [9]: existence of bone metastasis (bm), disease stage either 3 or 4 (stage), performance (pf), history of cardiovascular events (hx), age and weight (wt). We denote rx as treatment indicator, and include the two important interactions in the model, i.e. bm:rx and age:rx as in [9]. Table 3 provides the posterior mean and posterior standard deviation of the coefficients. We found that stage and age are not significant at a nominal 95% credible level, but we have a strong interaction with treatment of bone metastasis, and age.

**Table 3:** Posterior summaries of coefficients of covariates in prostate cancer dataset. The * indicates that the estimates are greater than 1.96 standard errors from 0. This is equivalent to 95% level.

Effect	Posterior Mean	Posterior SD	Significance
bm	0.575	1.170	*
stage	-0.012	0.470	
pf	0.178	0.773	*
hx	0.191	0.608	*
age	-0.021	0.018	
weight	-0.017	-0.001	*
rx	-6.085	-1.838	*
bm:rx	-1.125	-0.291	*
age:rx	0.026	0.082	*

**Fig 2:**
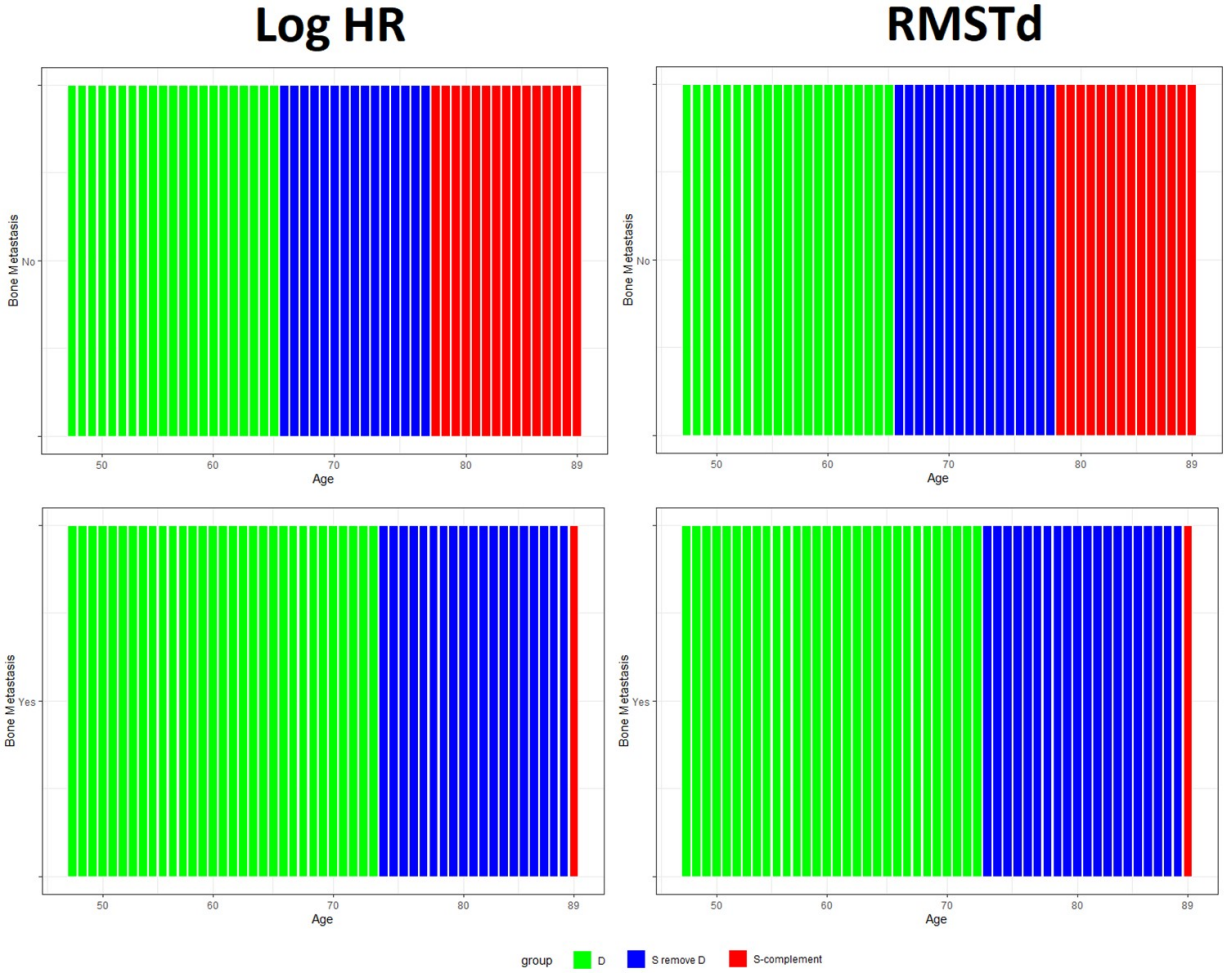
The Bayesian credible subgroups for prostate cancer by using Δ_*H*_ (left panel) and Δ_*Rd*_ (right panel) with credible level 95%.

The left panel in Fig 2 shows credible subgroups, for prostate cancer patients, using a log HR and a credible level of 95%. We used the same value *δ*_*H*_ = 1 to define subgroups ([9]). Each bar in each panel represents a particular type of patient with their age and existence of bone metastasis. Members in the green region (D) are benefiting from the treatment. The blue uncertainty region (*S*\*D*) contains characteristics of patients who are or are not be benefiting from the treatment. The red region (*S*^*C*^) indicates that these types of patients may not be benefiting from the treatment.

Similarly, the right panel in Fig 2 shows credible subgroups using the RMSTd with a credible level of 95% and *δ*_*R*_ = 0. Notice that the uncertainty region of Δ_*Rd*_ is smaller than the uncertainty region of Δ_*H*_ in patients with existence of bone metastasis. For both summaries, we found that when patients were younger than 67 years old and did not have any bone metastases, are benefiting to the treatment. Compared with the analysis reported by Ballarini et al. [9], the estimated benefiting and uncertainty regions are similar to our regions. However, our method, for both PTE approaches (Δ_*H*_ and Δ_*Rd*_), identified that patients who were older than 89 years and did not have existence of any bone metastases, may not be benefiting from the treatment. The PITE method did not identify these non–benefiting patients.

## 5 Analysis of a large simulated clinical trial dataset

We now illustrate our proposed methods on a simulated dateset based on a study published by Scirica et al. [22]. It is the Thrombin Receptor Antagonist in Secondary Prevention of Atherothrombotic Ischemic Events-Thrombolysis in Myocardial Infarction 50 trial. The primary efficacy endpoint is the time of first myocardial infarction, stroke or cardiovascular death. Even though patients with a history of myocardial infarction are treated for secondary prevention, they are still at risk of a recurrent thrombotic events. To reduce recurrent thrombotic events, patients are often treated with platelet inhibitors in addition to aspirin for up to a year, but this treatment also increases bleeding.

Scirica et al. [22] analyzed the vorapaxar dataset using a HR from a Cox proportional hazards model for testing heterogeneous effect across the prespecified subgroups of interest. In contrast to their approach, our aim is to search for benefiting subgroups without prespecifying subgroups of interest. To illustrate our proposed approach, we derived a simulated dataset from the proprietary dataset used by Scirica et al [22] in the following section.

### 5.1 Simulated dataset based on a large clinical trial dataset

The simulated data mimics the dataset presented at Scirica et al. [22] that pertains to 17,779 patients of whom 8898 were assigned to treatment and 8881 were assigned to placebo. We considered a 5-dimensional prognostic covariate vector which represented patients’ characteristics at baseline: age at entry (years), baseline weight (kilograms), history of hyperlipidemia, smoking status and prior coronary revascularization. For each treatment group, we randomly selected 20% of subjects and added a Gaussian noise with zero mean and standard deviation of 1 and 5 for continuous covariates age and baseline weight, respectively. Previous studies [36, 37] found that patients who are younger than 75 years old, with no history of stroke and bodyweight at least 60 kg are likely benefiting from the antiplatelet therapy. Hence we include age, baseline weight and history of prior coronary revascularization as predictive covariates.

**Fig 3:**
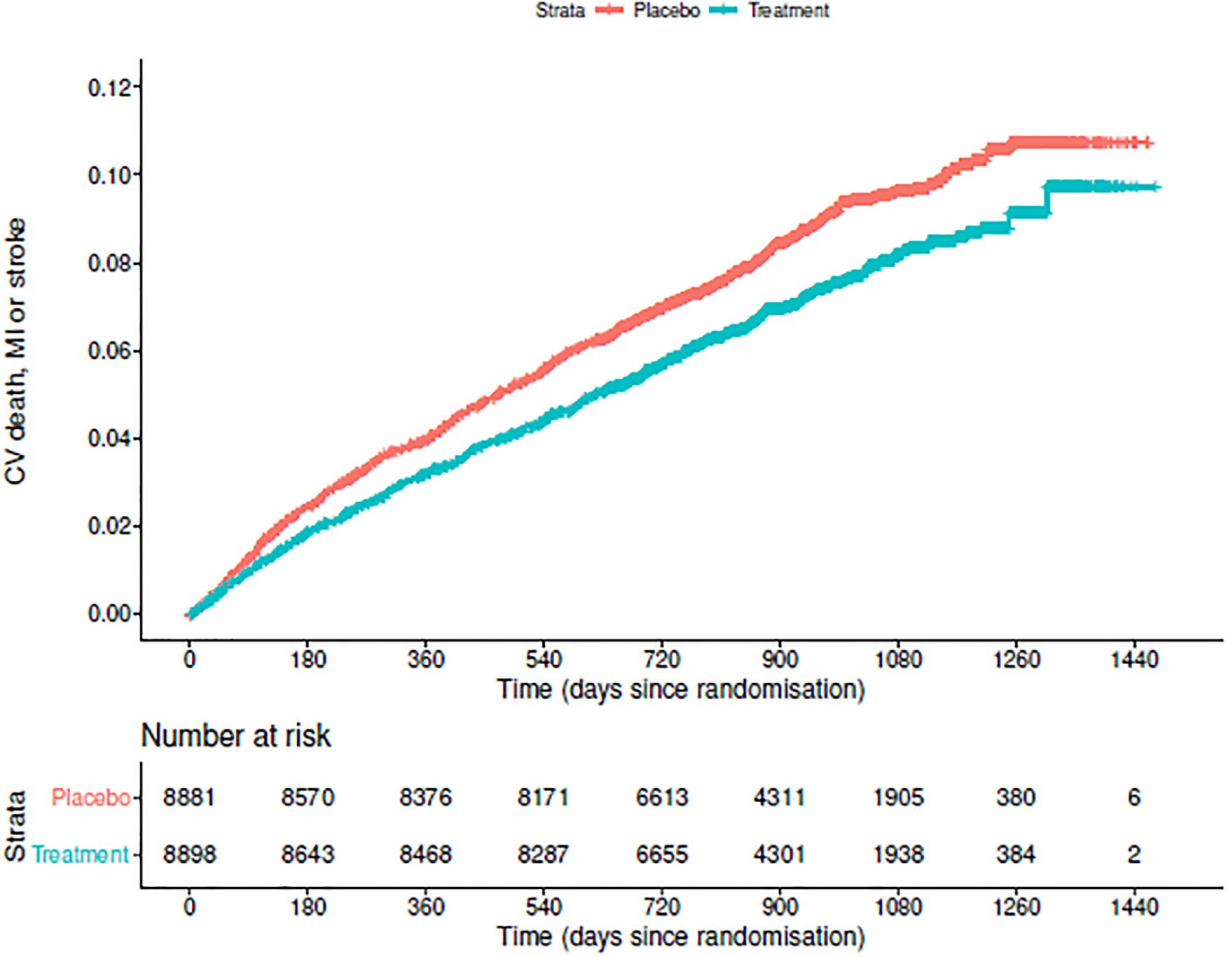
The Kaplan–Meier curve for the time of first myocardial infarction, stroke, or cardiovascular death for the simulated dataset.

The baseline characteristics of 17,779 subjects are summarized in Table 4. The median follow–up was 2.5 years (IQR 2–2.9 years), and the Kaplan–Meier curve [38] of estimated occurrence of the cardiovascular death, myocardial infarction or stroke is showed in Fig 3. The chance of cardiovascular death, myocardial infarction, or stroke was lower in patients in treatment group than those in placebo group over the follow–up time. Moreover, the global test of proportional hazards [39] fails to reject the assumption of proportional hazards (p–value = 0.51).

**Table 4:** Summary of baseline characteristics for simulated clinical trial dataset. We report the median with the first and third quartiles for continuous variables, and total count with its percent of the total trial population for categorical variables.

	Treatment (*n* = 8898)	Placebo (*n* = 8881)
Age (in years)	59 (52-66)	59 (52-66)
Weight (in kg)	85 (73.5-96)	85 (73-95.5)
Hyperlipidaemia	7568 (85%)	7545 (85%)
Smoking	1729 (19.4%)	1755 (%19.8)
Previous coronary revascularisation	7629 (85.7%)	7645 (86%)

### 5.2 Results

**Fig 4:**
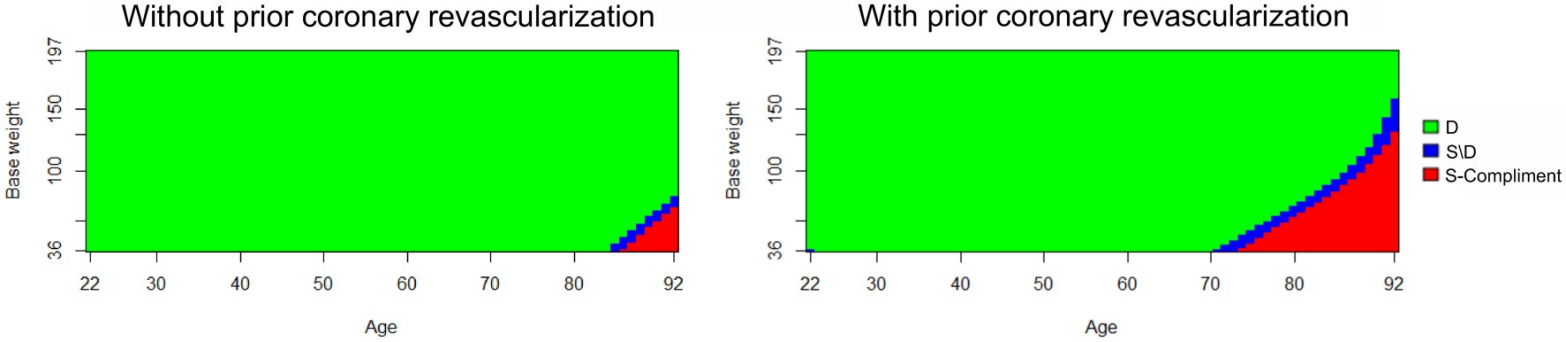
The Bayesian credible subgroups for the simulated dataset by using Δ_*H*_ with credible level 95%.

**Fig 5:**
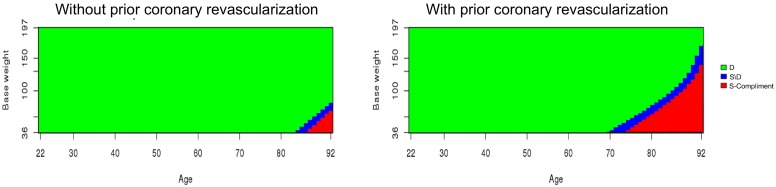
The Bayesian credible subgroups for the simulated dataset by using Δ_*Rd*_ with credible level 95%.

We applied Bayesian credible subgroup analysis to the simulated dataset using log HR and RMSTd as described in Section 2. Moreover, we applied cubic B-splines with three degrees of freedom due to their numerical stability [40] for continuous covariates: age at entry and baseline weight, and we chose one knot at medians of these covariates. Fig 4 presents the credible subgroups using Δ_*H*_ with credible level at 95% and *δ*_*H*_ = 1, and Fig 5 illustrates the credible subgroups using Δ_*Rd*_ with the same credible level at 95% and *δ*_*R*_ = 0. For subjects without history of prior coronary revascularization, the results of Δ_*H*_ and Δ_*Rd*_ are similar. Both approaches determine that types of patients younger than 82 years old and with bodyweight at least 80 kg benefit from the treatment versus the control. Moreover, we also have enough evidence to identify non–benefiting subgroup including types of patients who are older than 90 years and have bodyweight less than 78 kg.

For subjects with history of prior coronary revascularization, we found that the two approaches Δ_*H*_ and Δ_*Rd*_ yield similar credible subgroups except that the uncertainty region is slightly larger when using RMSTd. Both approaches find that types of patients aged older than 70 years, with history of prior coronary revascularization, and bodyweight at most 150 kg are not benefiting from a treatment. Note that there is relatively small uncertainty region around age of 22 for log HR approach and not for RMSTd approach. For both dataset, a comparison between the proposed Bayesian credible subgroup with the pointwise method are also reported in S1 File.

## 6 Discussion

We have presented a Bayesian credible subgroup method for survival endpoints by using two common summaries: log HR and RMSTd. Our proposed methods perform well in simulation studies with respect to frequentist properties for finding credible subgroup pairs *D* and *S*, such as a total coverage, and sensitivity and specificity of *D*. As shown in previous studies [17–20], compared to HR, RMST is a robust and clinically interpretable measure of the survival time distribution without PH assumption. We also demonstrated that an RMSTd approach is appropriate to identify benefiting subgroups for studies with nonproportional hazards. From our applications in the prostate cancer dataset and the simulated large clinical trial data, a Bayesian credible subgroup method, using two common summaries, can identify all member of exclusive subgroup *D* who benefit from a treatment, and non–benefiting subjects who are not members of inclusive subgroup *S*. Moreover, our proposed methods control multiplicity issues in contrast to previous studies.

The major advantage of the Bayesian framework is that it allows us to compute the joint posterior sample of the PTEs and assess the treatment effect across the covariate space. Due to the two–stage approach, our semi–parametric model (Cox proportional hazard regression) in the regression stage can be extended to parametric (accelerated failure time AFT regression) or non–parametric (Dirichlet process priors [41] or Bayesian additive regression trees (BART) [34]). The model choice depends on the flexibility and applicability necessary for the problem as long as the joint posterior sample of the PTEs can be obtained from the model. When using the parametric and semi-parametric model, it would be worth to further investigate the performance of credible subgroups in a case of model misspecification where neither AFT or PH assumption holds.

The first stage of our procedure requires only the list of possible covariates. However, a large number of predictive, especially continuous, covariates makes interpretation of the shape of credible subgroups difficult and reduces power. A possible approach and a topic for future research is to employ variable selection methods in the regression stage such as Bayesian lasso. Another approach is to compute the maximum credible level at which the test of no effect for a given subject’s covariate profile is rejected [15].

The methods we proposed in this article focus on a single efficacy endpoint in a clinical trial, but it can be generalized to include more than one endpoint [16]. Multiple efficacy and safety endpoints may be considered simultaneously to establish the actual estimated benefit-risk balance patients may experience depending on their individual characteristics. However, there may be some complexity around the choice of benefit-risk metrics used in combination with the credible subgroup method due to their level of discriminatory abilities [42–44]. Additionally, the endpoints that matter in a decision may also be in different units of measurement, and although the methods of utilities have been proposed as a potential solution, there may be other methodological issues. For example, the uncertainties relating to utilities are more complex to derive. Utilities are also a very specific concept that is context–specific, and may not be intuitive to the general public and decision–makers. Furthermore, there has been some shift in the pharmaceutical industry and regulatory focus on better use of patient preferences data in benefit–risk assessment, as evident by various global initiatives (IMI-PREFER [45, 46], PDUFA VI [47], FDA MDIC [48], PFDD [49]). Patient preferences and perspectives on certain outcomes or treatment options add a unique complexity to the problem because of the heterogeneous nature of patients. It is possible that patients with different characteristics, not only may respond differently to treatments but, may also have different preference values. It is not entirely clear at this time how preferences should be taken into account in relation to patient subgroups. Nevertheless, decisions about a benefit-risk balance of a treatment option must be made in the context of its benefits and risks, as well as patient preferences; and this presents a wealth of research opportunity to improve decision-making in healthcare.

Finally, our proposed methods in this article only address the scenario when there is no missing value in patients’ covariate. Although such case has not been investigated here, regression tree, e.g. BART, can be a potential approach for handling missing data without selection of imputation method [50]. As a closing remark, our Bayesian credible subgroup method for survival endpoint has a broad application in clinical trials as we demonstrated the method in two time–to–event dataset where identifying benefiting subgroups are important in discovering personalized treatment.

## Supporting information

S1 File Supplementary material for Bayesian credible subgroup identication for treatment effectiveness in time–to–event data. (PDF)
