# Supplementary material for: Bayesian credible subgroup identification for treatment effectiveness in time-to-event data
Source: PLoS One. 2020 Feb 26;15(2):e0229336. doi: 10.1371/journal.pone.0229336 (PMC7043747; doi:10.1371/journal.pone.0229336)
Supplement: S1 File — (PDF) [file pone.0229336.s001.pdf]

Supplementary Material for Bayesian Credible Subgroup Identification for Treatment Effectiveness in Time–to–event Data

Duy Ngo[1,3], Richard Baumgartner[1], Shahrul Mt-Isa[2,4], Dai Feng[1], Jie Chen[1], and Patrick Schnell[5]

[1]Merck & Co., Inc., Kenilworth, New Jersey, United States,
[2]MSD Research Laboratories, MSD, London, United Kingdom,
[3]Department of Statistics, Western Michigan University, Kalamazoo, Michigan, United States,
[4]School of Public Health, Imperial College London, London, United Kingdom,
[5]The Ohio State University College of Public Health, Columbus, Ohio, United States.

January 12, 2020

Contents

1 Background on Survival Analysis **2**

2 Relationship of log hazard ratio (log HR) and a difference in restricted mean survival time (RMSTd) **2**

3 Algorithms for constructing the posterior Δ_H and Δ_{Rd} **3**
3.1 Algorithms for constructing the posterior Δ_H . . . 3
3.2 Algorithms for constructing the posterior Δ_{Rd} . . . 3

4 Simulation study under proportional hazard assumption **3**
4.1 Simulation 1: both log HR and RMSTd. . . . 3
4.2 Simulation 2: for log HR. . . . 6
4.3 Simulation 3: for RMST differences. . . . 11

5 Model diagnosis **15**
5.1 The prostate cancer dataset . . . 15
5.2 A large simulated clinical trial dataset . . . 15

6 Pointwise method **17**
6.1 Simulation study . . . 17
6.2 Prostate cancer dataset . . . 17
6.3 A large simulated clinical trial dataset . . . 18

7 Simulation study under nonproportional hazard assumption **19**

1 Background on Survival Analysis

Let T be the time until some specified event, the cumulative distribution function $F(t)$ is defined as

$$F(t) = P(T \leq t).$$

Thus, the survival function (curve) $S(t)$ is defined to be the complementary function of $F(t)$ and expressed as

$$S(t) = 1 - F(t) = P(T > t),$$

which is the probability of an individual surviving beyond time t. When T is a continuous random variable, the survival function is the integral of the probability density function, $f(t)$, that is,

$$S(t) = P(T > t) = \int_t^\infty f(x)\, dx.$$

When T is a discrete random variable, the survival function for a discrete random variable T is given by

$$S(t) = P(T > t) = \sum_{t_j > t} p(t_j),$$

where t_j for $j = 1, 2, \ldots$ are possible values T can take, and $p(t_j) = P(T = t_j)$ where $t_1 < t_2 < \ldots$. Note that the survival function is a non–increasing step function.

2 Relationship of log hazard ratio (log HR) and a difference in restricted mean survival time (RMSTd)

For each subject $i = 1, \ldots, n$, let x_i and z_i be $p \times 1$ and $q \times 1$ vectors of prognostic and predictive covariates respectively. Furthermore, let $\theta_i = \{0, 1\}$ be the treatment indicator, i.e. $\theta_i = 1$ indicates subject i^{th} receives treatment. A Cox proportional hazards model assumes that

$$\lambda_i(t|x_i, z_i, \theta_i) = \lambda_0(t) e^{x_i^T \beta + \theta_i z_i^T \gamma}$$

where β and γ are $p \times 1$ and $q \times 1$ vectors of regression coefficients, and $\lambda_0(t) > 0$ is the baseline hazard function.

It follows that the survival function can be expressed as

$$S_i(t|x_i, z_i, \theta_i) = [S_0(t)]^{\exp\left(x_i^T \beta + \theta_i z_i^T \gamma\right)}$$

where $S_0(t) = e^{-\int_0^t \lambda_0(u) du}$ is the baseline survival function. For a particular subject ith, the personlized treatment effects (PTEs) as HR is defined as

$$\Delta_{H,i} = \frac{\lambda_i(t|x_i, z_i, \theta_i = 1)}{\lambda_i(t|x_i, z_i, \theta_i = 0)} = e^{z_i^T \gamma},$$

and the PTEs as RMSTd up to time $t^* > 0$ is expressed as

$$\Delta_{Rd,i} = \int_0^{t^*} S_i(t|x_i, z_i, \theta_i = 1) dt - \int_0^{t^*} S_i(t|x_i, z_i, \theta_i = 0) dt$$

$$\iff \Delta_{Rd,i} = \int_0^{t^*} \left([S_0(t)]^{\exp\left(x_i^T \beta + z_i^T \gamma\right)} - [S_0(t)]^{\exp\left(x_i^T \beta\right)} \right) dt$$

$$\iff \Delta_{Rd,i} = \int_0^{t^*} \left([S_0(t)]^{\exp\left(x_i^T \beta\right) \Delta_{H,i}} - [S_0(t)]^{\exp\left(x_i^T \beta\right)} \right) dt$$

It shows that when $\Delta_{H,i} = 1$ or $\log \Delta_{H,i} = 0$, we have $\Delta_{Rd,i} = 0$. Therefore, we set $\delta_H = 1$ and $\delta_{Rd} = 0$ in our simulation studies so that we can compare performance of these approaches for finding credible subgroup pairs.

3 Algorithms for constructing the posterior Δ_H and Δ_{Rd}

3.1 Algorithms for constructing the posterior Δ_H

As defined in Section 2.2 from the manuscript, the hazard ratio (HR) as a PTE for a patient with covariate z is

$$\Delta_H(z_i) = \frac{\lambda(t|x_i, z_i, \theta_i = 1)}{\lambda(t|x_i, z_i, \theta_i = 0)} = \exp(z_i'\gamma),$$

which is a ratio between the hazards of a patient with treatment $\theta = 1$ and $\theta = 0$. Alternatively, $\log \Delta_H(z_i) = z_i'\gamma$ is the log HR evaluated at points z_i. Following the notation in Section 2.4.2, the algorithm for constructing the posterior of Δ_H is presented below

Algorithm 1 Constructing the posterior Δ_H

Construct the covariate space grid Θ for z.
Initialize $\boldsymbol{\beta}_{(0)} = (\beta'_{(0)}, \gamma'_{(0)})'$, $\mathbf{h}_{(0)}$.
for $\ell = 1, 2, \ldots$ **do**
  $P(\beta_{i,(\ell)} \mid \boldsymbol{\beta}^{(-i)}_{(\ell-1)}, \mathbf{h}_{(\ell-1)}, \mathcal{D}) \propto \prod_{j=1}^{J} l_{j,(\ell-1)} \exp\left(\frac{-1}{2}(\boldsymbol{\beta}_{(\ell-1)} - \mu_0)\Sigma_0^{-1}(\boldsymbol{\beta}_{(\ell-1)} - \mu_0)\right).$
  $P(h_{j,(\ell)} \mid \mathbf{h}^{(-j)}, \boldsymbol{\beta}, \mathcal{D}) \propto h_{j,(\ell-1)}^{\alpha_j - \alpha_{j-1} - 1} \exp\left[-h_{j,(\ell-1)}\left(\sum_{k \in R_j \setminus M_j} \exp(\mathbf{x}_k'\boldsymbol{\beta}_{(\ell-1)}) + b\right)\right].$
end for
For each covariate point $z_i \in \Theta$, compute the posterior $\Delta_H(z_i)_{(\ell)} = \exp(z_i'\gamma_{(\ell)})$ or $\log \Delta_H(z_i) = z_i'\gamma_{(\ell)}$ (after remove number of burn–in iterations) .

3.2 Algorithms for constructing the posterior Δ_{Rd}

From Section 2.3 in the manuscript, the RMST difference between two arms from $t = 0$ to $t = \nu$ is defined as

$$\Delta_{Rd} = \psi_{\theta=1} - \psi_{\theta=0} = \int_0^{\nu} \left[S(t|\theta = 1) - S(t|\theta = 0)\right] dt.$$

which is the difference in area between the two survival curves. Then we employ the conventional Cox proportional hazard model to estimate these two survival functions. Following the notation in Section 2.4.2, the algorithm for constructing the posterior of Δ_{Rd} is presented below

Algorithm 2 Constructing the posterior Δ_{Rd}

Construct the covariate space grid Θ_0 for $\mathbf{x} = \{x, z\}$ and $\theta = 0$.
Set $\Theta_1 = \Theta_0$ and replace θ by $1 - \theta$.
Initialize $\boldsymbol{\beta}_{(0)} = (\beta'_{(0)}, \gamma'_{(0)})'$, $\mathbf{h}_{(0)}$.
for $\ell = 1, 2, \ldots$ **do**
  $P(\beta_{i,(\ell)} \mid \boldsymbol{\beta}^{(-i)}_{(\ell-1)}, \mathbf{h}_{(\ell-1)}, \mathcal{D}) \propto \prod_{j=1}^{J} l_{j,(\ell-1)} \exp\left(\frac{-1}{2}(\boldsymbol{\beta}_{(\ell-1)} - \mu_0)\Sigma_0^{-1}(\boldsymbol{\beta}_{(\ell-1)} - \mu_0)\right).$
  $P(h_{j,(\ell)} \mid \mathbf{h}^{(-j)}, \boldsymbol{\beta}, \mathcal{D}) \propto h_{j,(\ell-1)}^{\alpha_j - \alpha_{j-1} - 1} \exp\left[-h_{j,(\ell-1)}\left(\sum_{k \in R_j \setminus M_j} \exp(\mathbf{x}_k'\boldsymbol{\beta}_{(\ell-1)}) + b\right)\right].$
end for
Compute $H_{0,(\ell)}(t) = \sum_{j \leq t} h_{j,(l)}$.
For each $\mathbf{x}_k \in \Theta_0$, compute $S_{k,(\ell)}(t|\theta = 0) = \exp(-H_{0,(\ell)}(t)\exp(\mathbf{x}_k'\boldsymbol{\beta}_{(\ell)}))$.
For each $\mathbf{x}_k \in \Theta_1$, compute $S_{k,(\ell)}(t|\theta = 1) = \exp(-H_{0,(\ell)}(t)\exp(\mathbf{x}_k'\boldsymbol{\beta}_{(\ell)}))$.
Compute $\Delta_{Rd,k,(\ell)} = \int_0^{\nu}\left[S_{k,(\ell)}(t|\theta = 1) - S_{k,(\ell)}(t|\theta = 0)\right] dt.$

4 Simulation study under proportional hazard assumption

4.1 Simulation 1: both log HR and RMSTd.

For each simulation dataset, we evaluate the Bayesian credible subgroups method by using log HR and RMSTd. Moreover, we use the thresholds at 1 for HR and 0 for RMSTd to idetify benefiting subjects. Table 1–4 present the results of average summary statistics for different credible levels. The total coverage is always grater than the credible level. The credible pair size is decreasing when sample size and/or effective size are increasing. Sensitivity of D is higher when sample size is increasing. HR had higher sensitivity of D than RMSTd. However, both approaches have similar specificity of D. Lastly, RMSTd had smaller effect MSE than HR for large sample size ($n = 500, 1000$) and slightly larger than HR for smaller sample size ($n = 50, 100$). Table 1–4 show that, as expected, the two approaches had similar results.

Table 1: Average summary statistics for **40%** credible level.

Sample Size	Truth	Total Coverage		Credible Pair Size		Sensitivity of D		Specificity of D		MSE	
		log HR	RMST	log HR	RMST	log HR	RMST	log HR	RMST	log HR	RMST
50	(0,0,0,0,0)	0.62	0.6	0.74	0.75	NaN	NaN	0.88	0.89	0.44	0.48
	(0,0,0.1,0.1,0.1)	0.58	0.6	0.7	0.7	0.16	0.17	0.93	0.93	0.46	0.51
	(0,0,1,1,1)	0.66	0.66	0.18	0.18	0.7	0.69	0.97	0.97	0.46	0.38
	(0,0,1,-1,3)	0.66	0.62	0.06	0.05	0.99	0.99	0.96	0.95	0.96	0.28
	(0.2,0.2,1,1,1)	0.66	0.66	0.17	0.17	0.72	0.73	0.96	0.96	0.43	0.36
	(1,-2,1,0.1,1)	0.72	0.72	0.17	0.19	0.79	0.73	0.97	0.97	0.59	0.26
100	(0,0,0,0,0)	0.6	0.58	0.78	0.77	NaN	NaN	0.88	0.87	0.18	0.25
	(0,0,0.1,0.1,0.1)	0.63	0.61	0.69	0.68	0.24	0.25	0.93	0.94	0.2	0.26
	(0,0,1,1,1)	0.66	0.64	0.11	0.11	0.89	0.88	0.96	0.96	0.22	0.22
	(0,0,1,-1,3)	0.66	0.66	0.03	0.03	1	1	0.96	0.96	0.49	0.21
	(0.2,0.2,1,1,1)	0.66	0.65	0.11	0.11	0.89	0.88	0.96	0.96	0.23	0.21
	(1,-2,1,0.1,1)	0.74	0.74	0.1	0.11	0.94	0.92	0.97	0.97	0.28	0.2
500	(0,0,0,0,0)	0.6	0.63	0.78	0.79	NaN	NaN	0.88	0.89	0.03	0.05
	(0,0,0.1,0.1,0.1)	0.68	0.67	0.5	0.49	0.28	0.29	0.97	0.97	0.03	0.05
	(0,0,1,1,1)	0.68	0.64	0.08	0.08	1	1	0.96	0.96	0.08	0.06
	(0,0,1,-1,3)	0.68	0.72	0.03	0.03	1	1	0.96	0.96	0.31	0.05
	(0.2,0.2,1,1,1)	0.73	0.68	0.09	0.08	1	1	0.97	0.96	0.08	0.06
	(1,-2,1,0.1,1)	0.7	0.68	0.07	0.06	1	1	0.97	0.97	0.07	0.07
1000	(0,0,0,0,0)	0.68	0.66	0.81	0.79	NaN	NaN	0.91	0.9	0.01	0.03
	(0,0,0.1,0.1,0.1)	0.7	0.66	0.37	0.35	0.42	0.42	0.97	0.97	0.02	0.03
	(0,0,1,1,1)	0.62	0.6	0.08	0.08	1	1	0.95	0.95	0.06	0.03
	(0,0,1,-1,3)	0.64	0.6	0.03	0.02	1	1	0.96	0.95	0.28	0.03
	(0.2,0.2,1,1,1)	0.62	0.64	0.08	0.08	1	1	0.95	0.95	0.07	0.04
	(1,-2,1,0.1,1)	0.74	0.72	0.06	0.06	1	1	0.97	0.97	0.05	0.04

Table 2: Average summary statistics for **60%** credible level.

Sample Size	Truth	Total Coverage		Credible Pair Size		Sensitivity of D		Specificity of D		MSE	
		log HR	RMST	log HR	RMST	log HR	RMST	log HR	RMST	log HR	RMST
50	(0,0,0,0,0)	0.76	0.76	0.86	0.86	NaN	NaN	0.94	0.94	0.44	0.48
	(0,0,0.1,0.1,0.1)	0.74	0.76	0.83	0.85	0.1	0.09	0.96	0.96	0.46	0.51
	(0,0,1,1,1)	0.8	0.83	0.25	0.26	0.57	0.58	0.98	0.98	0.46	0.38
	(0,0,1,-1,3)	0.81	0.82	0.08	0.09	0.99	0.98	0.98	0.98	0.96	0.28
	(0.2,0.2,1,1,1)	0.81	0.84	0.23	0.27	0.62	0.61	0.98	0.98	0.43	0.36
	(1,-2,1,0.1,1)	0.87	0.89	0.25	0.33	0.68	0.5	0.99	0.99	0.59	0.26
100	(0,0,0,0,0)	0.73	0.74	0.87	0.87	NaN	NaN	0.93	0.93	0.18	0.25
	(0,0,0.1,0.1,0.1)	0.73	0.75	0.81	0.8	0.15	0.15	0.96	0.97	0.2	0.26
	(0,0,1,1,1)	0.8	0.78	0.15	0.16	0.83	0.82	0.98	0.98	0.22	0.22
	(0,0,1,-1,3)	0.8	0.76	0.05	0.05	1	1	0.98	0.97	0.49	0.21
	(0.2,0.2,1,1,1)	0.77	0.77	0.15	0.15	0.82	0.82	0.97	0.97	0.23	0.21
	(1,-2,1,0.1,1)	0.89	0.86	0.15	0.16	0.89	0.86	0.99	0.99	0.28	0.2
500	(0,0,0,0,0)	0.73	0.77	0.88	0.88	NaN	NaN	0.93	0.94	0.03	0.05
	(0,0,0.1,0.1,0.1)	0.84	0.84	0.63	0.63	0.18	0.18	0.99	0.99	0.03	0.05
	(0,0,1,1,1)	0.8	0.8	0.11	0.11	1	1	0.98	0.98	0.08	0.06
	(0,0,1,-1,3)	0.8	0.79	0.04	0.04	1	1	0.97	0.97	0.31	0.05
	(0.2,0.2,1,1,1)	0.82	0.8	0.11	0.1	1	1	0.98	0.98	0.08	0.06
	(1,-2,1,0.1,1)	0.84	0.8	0.1	0.09	1	1	0.98	0.98	0.07	0.07
1000	(0,0,0,0,0)	0.8	0.8	0.9	0.9	NaN	NaN	0.95	0.95	0.01	0.03
	(0,0,0.1,0.1,0.1)	0.84	0.82	0.47	0.46	0.29	0.29	0.99	0.98	0.02	0.03
	(0,0,1,1,1)	0.77	0.72	0.11	0.1	1	1	0.97	0.97	0.06	0.03
	(0,0,1,-1,3)	0.75	0.74	0.05	0.04	1	1	0.97	0.97	0.28	0.03
	(0.2,0.2,1,1,1)	0.78	0.76	0.11	0.1	1	1	0.97	0.97	0.07	0.04
	(1,-2,1,0.1,1)	0.84	0.83	0.09	0.09	1	1	0.98	0.98	0.05	0.04

Table 3: Average summary statistics for **80%** credible level.

Sample Size	Truth	Total Coverage		Credible Pair Size		Sensitivity of D		Specificity of D		MSE	
		log HR	RMST	log HR	RMST	log HR	RMST	log HR	RMST	log HR	RMST
50	(0,0,0,0,0)	0.88	0.88	0.94	0.93	NaN	NaN	0.97	0.97	0.44	0.48
	(0,0,0.1,0.1,0.1)	0.9	0.86	0.94	0.93	0.04	0.04	0.99	0.98	0.46	0.51
	(0,0,1,1,1)	0.92	0.94	0.32	0.38	0.45	0.47	0.99	0.99	0.46	0.38
	(0,0,1,-1,3)	0.92	0.94	0.12	0.19	0.97	0.97	0.99	0.99	0.96	0.28
	(0.2,0.2,1,1,1)	0.9	0.92	0.31	0.45	0.45	0.44	0.99	0.99	0.43	0.36
	(1,-2,1,0.1,1)	0.96	0.97	0.35	0.55	0.52	0.12	1	1	0.59	0.26
100	(0,0,0,0,0)	0.9	0.89	0.95	0.95	NaN	NaN	0.98	0.98	0.18	0.25
	(0,0,0.1,0.1,0.1)	0.89	0.91	0.9	0.91	0.08	0.08	0.99	0.99	0.2	0.26
	(0,0,1,1,1)	0.92	0.92	0.21	0.21	0.72	0.73	0.99	0.99	0.22	0.22
	(0,0,1,-1,3)	0.91	0.92	0.08	0.08	1	1	0.99	0.99	0.49	0.21
	(0.2,0.2,1,1,1)	0.89	0.9	0.21	0.22	0.72	0.71	0.99	0.99	0.23	0.21
	(1,-2,1,0.1,1)	0.96	0.96	0.21	0.3	0.79	0.57	1	1	0.28	0.2
500	(0,0,0,0,0)	0.88	0.9	0.95	0.95	NaN	NaN	0.97	0.98	0.03	0.05
	(0,0,0.1,0.1,0.1)	0.94	0.92	0.77	0.77	0.08	0.08	1	1	0.03	0.05
	(0,0,1,1,1)	0.9	0.92	0.13	0.13	1	1	0.99	0.99	0.08	0.06
	(0,0,1,-1,3)	0.88	0.86	0.06	0.05	1	1	0.98	0.98	0.31	0.05
	(0.2,0.2,1,1,1)	0.94	0.92	0.13	0.13	1	1	0.99	0.99	0.08	0.06
	(1,-2,1,0.1,1)	0.92	0.92	0.12	0.12	1	1	0.99	0.99	0.07	0.07
1000	(0,0,0,0,0)	0.9	0.89	0.97	0.96	NaN	NaN	0.98	0.98	0.01	0.03
	(0,0,0.1,0.1,0.1)	0.94	0.94	0.59	0.58	0.15	0.18	1	0.99	0.02	0.03
	(0,0,1,1,1)	0.88	0.88	0.13	0.13	1	1	0.99	0.99	0.06	0.03
	(0,0,1,-1,3)	0.87	0.88	0.06	0.06	1	1	0.98	0.98	0.28	0.03
	(0.2,0.2,1,1,1)	0.86	0.89	0.12	0.13	1	1	0.98	0.99	0.07	0.04
	(1,-2,1,0.1,1)	0.93	0.93	0.11	0.11	1	1	0.99	0.99	0.05	0.04

Table 4: Average summary statistics for **95%** credible level.

Sample Size	Truth	Total Coverage		Credible Pair Size		Sensitivity of D		Specificity of D		MSE	
		log HR	RMST	log HR	RMST	log HR	RMST	log HR	RMST	log HR	RMST
50	(0,0,0,0,0)	0.96	0.97	0.99	0.98	NaN	NaN	1	1	0.44	0.48
	(0,0,0.1,0.1,0.1)	0.96	0.96	0.99	0.98	0.01	0.03	1	0.99	0.46	0.51
	(0,0,1,1,1)	0.98	1	0.42	0.65	0.22	0.24	1	1	0.46	0.38
	(0,0,1,-1,3)	0.97	1	0.16	0.48	0.92	0.88	1	1	0.96	0.28
	(0.2,0.2,1,1,1)	0.97	0.99	0.41	0.78	0.26	0.19	1	1	0.43	0.36
	(1,-2,1,0.1,1)	1	1	0.5	0.74	0.32	0	1	1	0.59	0.26
100	(0,0,0,0,0)	0.98	0.98	0.99	0.99	NaN	NaN	1	0.99	0.18	0.25
	(0,0,0.1,0.1,0.1)	0.98	0.96	0.97	0.98	0.02	0.02	1	1	0.2	0.26
	(0,0,1,1,1)	0.97	1	0.29	0.36	0.51	0.49	1	1	0.22	0.22
	(0,0,1,-1,3)	0.97	1	0.11	0.21	0.99	1	1	1	0.49	0.21
	(0.2,0.2,1,1,1)	0.96	0.99	0.3	0.45	0.51	0.46	1	1	0.23	0.21
	(1,-2,1,0.1,1)	1	1	0.3	0.59	0.62	0.08	1	1	0.28	0.2
500	(0,0,0,0,0)	0.96	0.98	0.99	0.99	NaN	NaN	0.99	0.99	0.03	0.05
	(0,0,0.1,0.1,0.1)	1	0.98	0.88	0.89	0.01	0.02	1	1	0.03	0.05
	(0,0,1,1,1)	0.98	0.98	0.14	0.14	0.98	0.98	1	1	0.08	0.06
	(0,0,1,-1,3)	0.96	0.96	0.07	0.07	1	1	0.99	1	0.31	0.05
	(0.2,0.2,1,1,1)	0.98	0.98	0.14	0.14	0.98	0.97	1	1	0.08	0.06
	(1,-2,1,0.1,1)	0.96	0.96	0.13	0.14	1	1	1	1	0.07	0.07
1000	(0,0,0,0,0)	0.99	0.99	1	0.99	NaN	NaN	1	1	0.01	0.03
	(0,0,0.1,0.1,0.1)	0.98	0.98	0.74	0.74	0.05	0.05	1	1	0.02	0.03
	(0,0,1,1,1)	0.98	0.96	0.14	0.14	1	1	1	1	0.06	0.03
	(0,0,1,-1,3)	0.98	0.95	0.07	0.07	1	1	1	0.99	0.28	0.03
	(0.2,0.2,1,1,1)	0.96	0.98	0.14	0.14	1	1	1	1	0.07	0.04
	(1,-2,1,0.1,1)	1	0.98	0.13	0.13	1	1	1	1	0.05	0.04

4.2 Simulation 2: for log HR.

Here we evaluate the Bayesian credible subgroup by using log HR to identify the benefiting subjects. Table 5–8 show that total coverage is always greater than the credible level. When sample size is increasing, the credible pair size is decreasing, but the effect MSE is improving. Finally, the sensitivity and specificity of D is high.

Table 5: Average summary statistics for threshold $\delta = \mathbf{0.2}$. at 4 different credible levels.

Sample Size	Truth	Total Coverage				Credible Pair Size			
		40%	60%	80%	95%	40%	60%	80%	95%
50	(0,0,0,0,0)	0.99	1	1	1	0.04	0.09	0.19	0.43
	(0,0,0.1,0.1,0.1)	0.99	1	1	1	0.03	0.07	0.14	0.3
	(0,0,1,1,1)	0.74	0.88	0.94	0.99	0.1	0.14	0.19	0.25
	(0,0,1,-1,3)	0.86	0.93	0.97	1	0.05	0.07	0.11	0.17
	(0.2,0.2,1,1,1)	0.72	0.86	0.94	0.98	0.1	0.14	0.19	0.25
	(1,-2,1,0.1,1)	0.74	0.86	0.96	1	0.13	0.18	0.23	0.28
100	(0,0,0,0,0)	1	1	1	1	0	0	0.02	0.09
	(0,0,0.1,0.1,0.1)	1	1	1	1	0	0	0.01	0.05
	(0,0,1,1,1)	0.82	0.93	0.98	1	0.07	0.1	0.15	0.19
	(0,0,1,-1,3)	0.88	0.92	0.98	1	0.03	0.04	0.07	0.1
	(0.2,0.2,1,1,1)	0.82	0.9	0.96	0.98	0.07	0.1	0.14	0.19
	(1,-2,1,0.1,1)	0.78	0.86	0.96	0.99	0.1	0.14	0.18	0.23
500	(0,0,0,0,0)	1	1	1	1	0	0	0	0
	(0,0,0.1,0.1,0.1)	1	1	1	1	0	0	0	0
	(0,0,1,1,1)	0.94	0.97	0.99	1	0.02	0.03	0.04	0.08
	(0,0,1,-1,3)	0.96	0.98	1	1	0.02	0.02	0.03	0.05
	(0.2,0.2,1,1,1)	0.92	0.95	0.98	1	0.02	0.02	0.05	0.08
	(1,-2,1,0.1,1)	0.92	0.96	0.99	1	0.05	0.07	0.1	0.13
1000	(0,0,0,0,0)	1	1	1	1	0	0	0	0
	(0,0,0.1,0.1,0.1)	1	1	1	1	0	0	0	0
	(0,0,1,1,1)	0.96	1	1	1	0.01	0.02	0.03	0.05
	(0,0,1,-1,3)	0.99	1	1	1	0	0.01	0.02	0.03
	(0.2,0.2,1,1,1)	0.98	0.99	1	1	0.01	0.02	0.03	0.05
	(1,-2,1,0.1,1)	0.94	0.98	1	1	0.04	0.06	0.08	0.11

Sample Size	Truth	Sensitivity of D				Specificity of D			
		40%	60%	80%	95%	40%	60%	80%	95%
50	(0,0,0,0,0)	NaN	NaN	NaN	NaN	1	1	1	1
	(0,0,0.1,0.1,0.1)	NaN	NaN	NaN	NaN	1	1	1	1
	(0,0,1,1,1)	0.34	0.22	0.12	0.04	0.99	1	1	1
	(0,0,1,-1,3)	0.88	0.83	0.78	0.68	1	1	1	1
	(0.2,0.2,1,1,1)	0.38	0.24	0.1	0.03	0.99	1	1	1
	(1,-2,1,0.1,1)	0.26	0.17	0.08	0.03	1	1	1	1
100	(0,0,0,0,0)	NaN	NaN	NaN	NaN	1	1	1	1
	(0,0,0.1,0.1,0.1)	NaN	NaN	NaN	NaN	1	1	1	1
	(0,0,1,1,1)	0.44	0.26	0.12	0.04	1	1	1	1
	(0,0,1,-1,3)	0.92	0.89	0.84	0.78	1	1	1	1
	(0.2,0.2,1,1,1)	0.39	0.22	0.12	0.03	1	1	1	1
	(1,-2,1,0.1,1)	0.3	0.19	0.1	0.02	1	1	1	1
500	(0,0,0,0,0)	NaN	NaN	NaN	NaN	1	1	1	1
	(0,0,0.1,0.1,0.1)	NaN	NaN	NaN	NaN	1	1	1	1
	(0,0,1,1,1)	0.7	0.62	0.44	0.16	1	1	1	1
	(0,0,1,-1,3)	0.96	0.94	0.92	0.88	1	1	1	1
	(0.2,0.2,1,1,1)	0.7	0.62	0.37	0.16	1	1	1	1
	(1,-2,1,0.1,1)	0.57	0.48	0.28	0.12	1	1	1	1
1000	(0,0,0,0,0)	NaN	NaN	NaN	NaN	1	1	1	1
	(0,0,0.1,0.1,0.1)	NaN	NaN	NaN	NaN	1	1	1	1
	(0,0,1,1,1)	0.82	0.73	0.6	0.33	1	1	1	1
	(0,0,1,-1,3)	0.99	0.98	0.96	0.92	1	1	1	1
	(0.2,0.2,1,1,1)	0.82	0.7	0.52	0.26	1	1	1	1
	(1,-2,1,0.1,1)	0.68	0.56	0.4	0.21	1	1	1	1

Table 6: Average summary statistics for threshold $\delta = \mathbf{0.5}$ at 4 different credible levels.

Sample Size	Truth	Total Coverage				Credible Pair Size			
		40%	60%	80%	95%	40%	60%	80%	95%
50	(0,0,0,0,0)	0.88	0.94	0.98	1	0.44	0.6	0.75	0.92
	(0,0,0.1,0.1,0.1)	0.94	0.97	0.99	1	0.34	0.48	0.65	0.85
	(0,0,1,1,1)	0.79	0.88	0.95	1	0.17	0.22	0.28	0.35
	(0,0,1,-1,3)	0.95	0.98	1	1	0.03	0.06	0.09	0.15
	(0.2,0.2,1,1,1)	0.8	0.9	0.94	0.98	0.16	0.21	0.27	0.35
	(1,-2,1,0.1,1)	0.79	0.87	0.96	1	0.17	0.23	0.31	0.41
100	(0,0,0,0,0)	0.98	0.99	1	1	0.24	0.38	0.56	0.78
	(0,0,0.1,0.1,0.1)	0.98	0.99	1	1	0.13	0.2	0.35	0.61
	(0,0,1,1,1)	0.87	0.96	1	1	0.1	0.16	0.21	0.28
	(0,0,1,-1,3)	0.97	0.98	1	1	0.01	0.02	0.04	0.08
	(0.2,0.2,1,1,1)	0.86	0.92	0.96	1	0.11	0.16	0.21	0.29
	(1,-2,1,0.1,1)	0.81	0.9	0.97	0.99	0.11	0.15	0.21	0.3
500	(0,0,0,0,0)	1	1	1	1	0	0	0.01	0.08
	(0,0,0.1,0.1,0.1)	1	1	1	1	0.01	0.01	0.03	0.07
	(0,0,1,1,1)	0.94	0.98	1	1	0.04	0.06	0.09	0.12
	(0,0,1,-1,3)	1	1	1	1	0	0	0	0
	(0.2,0.2,1,1,1)	0.94	0.98	1	1	0.04	0.06	0.09	0.12
	(1,-2,1,0.1,1)	0.91	0.97	0.99	1	0.04	0.06	0.09	0.12
1000	(0,0,0,0,0)	1	1	1	1	0	0	0	0
	(0,0,0.1,0.1,0.1)	1	1	1	1	0	0	0	0.01
	(0,0,1,1,1)	0.98	1	1	1	0.02	0.04	0.06	0.1
	(0,0,1,-1,3)	1	1	1	1	0	0	0	0
	(0.2,0.2,1,1,1)	0.98	1	1	1	0.02	0.04	0.06	0.1
	(1,-2,1,0.1,1)	0.96	0.99	1	1	0.03	0.05	0.07	0.1

Sample Size	Truth	Sensitivity of D				Specificity of D			
		40%	60%	80%	95%	40%	60%	80%	95%
50	(0,0,0,0,0)	NaN	NaN	NaN	NaN	0.98	0.99	1	1
	(0,0,0.1,0.1,0.1)	NaN	NaN	NaN	NaN	0.99	1	1	1
	(0,0,1,1,1)	0.44	0.34	0.22	0.09	0.99	1	1	1
	(0,0,1,-1,3)	0.98	0.95	0.92	0.84	0.99	1	1	1
	(0.2,0.2,1,1,1)	0.46	0.34	0.22	0.09	1	1	1	1
	(1,-2,1,0.1,1)	0.48	0.36	0.22	0.1	1	1	1	1
100	(0,0,0,0,0)	NaN	NaN	NaN	NaN	1	1	1	1
	(0,0,0.1,0.1,0.1)	NaN	NaN	NaN	NaN	1	1	1	1
	(0,0,1,1,1)	0.58	0.45	0.34	0.18	1	1	1	1
	(0,0,1,-1,3)	1	0.99	0.98	0.94	1	1	1	1
	(0.2,0.2,1,1,1)	0.53	0.41	0.29	0.14	1	1	1	1
	(1,-2,1,0.1,1)	0.6	0.52	0.39	0.23	1	1	1	1
500	(0,0,0,0,0)	NaN	NaN	NaN	NaN	1	1	1	1
	(0,0,0.1,0.1,0.1)	NaN	NaN	NaN	NaN	1	1	1	1
	(0,0,1,1,1)	0.79	0.71	0.6	0.45	1	1	1	1
	(0,0,1,-1,3)	1	1	1	1	1	1	1	1
	(0.2,0.2,1,1,1)	0.78	0.71	0.58	0.44	1	1	1	1
	(1,-2,1,0.1,1)	0.83	0.77	0.68	0.57	1	1	1	1
1000	(0,0,0,0,0)	NaN	NaN	NaN	NaN	1	1	1	1
	(0,0,0.1,0.1,0.1)	NaN	NaN	NaN	NaN	1	1	1	1
	(0,0,1,1,1)	0.88	0.83	0.74	0.55	1	1	1	1
	(0,0,1,-1,3)	1	1	1	1	1	1	1	1
	(0.2,0.2,1,1,1)	0.9	0.81	0.7	0.52	1	1	1	1
	(1,-2,1,0.1,1)	0.88	0.83	0.74	0.65	1	1	1	1

Table 7: Average summary statistics for threshold $\delta = \mathbf{1}$.

Sample Size	Truth	Total Coverage				Credible Pair Size			
		40%	60%	80%	95%	40%	60%	80%	95%
50	(0,0,0,0,0)	0.58	0.76	0.87	0.96	0.77	0.89	0.96	0.99
	(0,0,0.1,0.1,0.1)	0.6	0.78	0.9	0.98	0.72	0.85	0.94	0.99
	(0,0,1,1,1)	0.68	0.79	0.9	0.97	0.17	0.23	0.32	0.42
	(0,0,1,-1,3)	0.7	0.82	0.93	0.98	0.05	0.08	0.11	0.15
	(0.2,0.2,1,1,1)	0.66	0.8	0.9	0.99	0.17	0.24	0.32	0.41
	(1,-2,1,0.1,1)	0.76	0.86	0.95	0.99	0.17	0.24	0.34	0.5
100	(0,0,0,0,0)	0.66	0.79	0.89	0.98	0.78	0.88	0.94	0.99
	(0,0,0.1,0.1,0.1)	0.66	0.82	0.94	0.98	0.72	0.85	0.94	0.98
	(0,0,1,1,1)	0.66	0.8	0.9	0.98	0.11	0.16	0.21	0.29
	(0,0,1,-1,3)	0.64	0.78	0.9	0.96	0.03	0.06	0.08	0.11
	(0.2,0.2,1,1,1)	0.73	0.84	0.94	1	0.12	0.16	0.22	0.3
	(1,-2,1,0.1,1)	0.74	0.85	0.96	0.98	0.1	0.15	0.21	0.3
500	(0,0,0,0,0)	0.53	0.7	0.84	0.94	0.73	0.84	0.93	0.98
	(0,0,0.1,0.1,0.1)	0.65	0.81	0.92	0.98	0.47	0.61	0.74	0.88
	(0,0,1,1,1)	0.61	0.74	0.88	0.96	0.07	0.1	0.12	0.14
	(0,0,1,-1,3)	0.7	0.8	0.9	0.94	0.03	0.04	0.06	0.06
	(0.2,0.2,1,1,1)	0.62	0.8	0.9	0.98	0.08	0.1	0.13	0.14
	(1,-2,1,0.1,1)	0.74	0.82	0.92	0.96	0.07	0.1	0.12	0.13
1000	(0,0,0,0,0)	0.6	0.79	0.9	0.98	0.75	0.87	0.94	0.99
	(0,0,0.1,0.1,0.1)	0.74	0.88	0.96	1	0.38	0.5	0.63	0.78
	(0,0,1,1,1)	0.64	0.76	0.89	0.97	0.08	0.1	0.12	0.14
	(0,0,1,-1,3)	0.58	0.7	0.84	0.96	0.03	0.04	0.06	0.07
	(0.2,0.2,1,1,1)	0.65	0.76	0.86	0.98	0.08	0.1	0.12	0.14
	(1,-2,1,0.1,1)	0.74	0.86	0.93	0.98	0.06	0.09	0.11	0.13

Sample Size	Truth	Sensitivity of D				Specificity of D			
		40%	60%	80%	95%	40%	60%	80%	95%
50	(0,0,0,0,0)	NaN	NaN	NaN	NaN	0.87	0.94	0.97	0.99
	(0,0,0.1,0.1,0.1)	0.16	0.1	0.04	0.01	0.94	0.97	0.99	1
	(0,0,1,1,1)	0.77	0.67	0.47	0.27	0.96	0.98	0.99	1
	(0,0,1,-1,3)	1	0.99	0.98	0.94	0.96	0.98	0.99	1
	(0.2,0.2,1,1,1)	0.76	0.64	0.48	0.27	0.96	0.98	0.99	1
	(1,-2,1,0.1,1)	0.8	0.69	0.53	0.32	0.97	0.99	1	1
100	(0,0,0,0,0)	NaN	NaN	NaN	NaN	0.9	0.95	0.97	1
	(0,0,0.1,0.1,0.1)	0.14	0.07	0.03	0.01	0.96	0.98	0.99	1
	(0,0,1,1,1)	0.94	0.84	0.74	0.56	0.96	0.98	0.99	1
	(0,0,1,-1,3)	1	1	1	0.99	0.95	0.97	0.99	1
	(0.2,0.2,1,1,1)	0.88	0.81	0.7	0.48	0.97	0.98	0.99	1
	(1,-2,1,0.1,1)	0.94	0.9	0.8	0.62	0.97	0.98	1	1
500	(0,0,0,0,0)	NaN	NaN	NaN	NaN	0.86	0.92	0.96	0.99
	(0,0,0.1,0.1,0.1)	0.33	0.21	0.12	0.02	0.96	0.98	0.99	1
	(0,0,1,1,1)	1	1	1	0.99	0.95	0.97	0.99	1
	(0,0,1,-1,3)	1	1	1	1	0.96	0.98	0.99	0.99
	(0.2,0.2,1,1,1)	1	1	1	0.99	0.95	0.98	0.99	1
	(1,-2,1,0.1,1)	1	1	1	1	0.97	0.98	0.99	1
1000	(0,0,0,0,0)	NaN	NaN	NaN	NaN	0.89	0.95	0.98	1
	(0,0,0.1,0.1,0.1)	0.38	0.25	0.13	0.03	0.97	0.99	1	1
	(0,0,1,1,1)	1	1	1	1	0.96	0.97	0.99	1
	(0,0,1,-1,3)	1	1	1	1	0.95	0.96	0.98	0.99
	(0.2,0.2,1,1,1)	1	1	1	1	0.96	0.97	0.99	1
	(1,-2,1,0.1,1)	1	1	1	1	0.97	0.98	0.99	1

Table 8: Average summary statistics for threshold $\delta = \mathbf{2}$.

Sample Size	Truth	Total Coverage				Credible Pair Size			
		40%	60%	80%	95%	40%	60%	80%	95%
50	(0,0,0,0,0)	0.94	0.98	1	1	0.48	0.63	0.78	0.92
	(0,0,0.1,0.1,0.1)	0.85	0.94	0.98	0.99	0.55	0.68	0.82	0.94
	(0,0,1,1,1)	0.78	0.9	0.96	0.98	0.16	0.23	0.32	0.45
	(0,0,1,-1,3)	0.84	0.92	0.97	0.99	0.06	0.08	0.11	0.15
	(0.2,0.2,1,1,1)	0.81	0.9	0.98	1	0.16	0.24	0.32	0.46
	(1,-2,1,0.1,1)	0.82	0.94	0.98	1	0.17	0.25	0.37	0.56
100	(0,0,0,0,0)	0.96	0.99	1	1	0.24	0.36	0.54	0.76
	(0,0,0.1,0.1,0.1)	0.96	0.98	0.99	1	0.39	0.55	0.72	0.88
	(0,0,1,1,1)	0.84	0.94	1	1	0.09	0.14	0.2	0.27
	(0,0,1,-1,3)	0.84	0.92	0.96	1	0.04	0.06	0.08	0.11
	(0.2,0.2,1,1,1)	0.86	0.95	0.99	1	0.1	0.15	0.21	0.29
	(1,-2,1,0.1,1)	0.88	0.96	0.99	1	0.09	0.14	0.21	0.31
500	(0,0,0,0,0)	1	1	1	1	0	0.01	0.01	0.06
	(0,0,0.1,0.1,0.1)	0.98	0.99	1	1	0.06	0.1	0.16	0.28
	(0,0,1,1,1)	0.96	0.99	1	1	0.03	0.05	0.07	0.11
	(0,0,1,-1,3)	0.94	0.96	0.98	1	0.02	0.03	0.04	0.06
	(0.2,0.2,1,1,1)	0.99	0.99	1	1	0.03	0.04	0.07	0.12
	(1,-2,1,0.1,1)	1	1	1	1	0.01	0.03	0.05	0.08
1000	(0,0,0,0,0)	1	1	1	1	0	0	0	0
	(0,0,0.1,0.1,0.1)	1	1	1	1	0.02	0.03	0.06	0.12
	(0,0,1,1,1)	1	1	1	1	0.01	0.02	0.04	0.07
	(0,0,1,-1,3)	0.98	0.98	1	1	0.01	0.02	0.03	0.04
	(0.2,0.2,1,1,1)	1	1	1	1	0.01	0.02	0.03	0.07
	(1,-2,1,0.1,1)	1	1	1	1	0.01	0.01	0.02	0.04

Sample Size	Truth	Sensitivity of D				Specificity of D			
		40%	60%	80%	95%	40%	60%	80%	95%
50	(0,0,0,0,0)	0.51	0.37	0.22	0.08	NaN	NaN	NaN	NaN
	(0,0,0.1,0.1,0.1)	0.43	0.31	0.18	0.06	NaN	NaN	NaN	NaN
	(0,0,1,1,1)	0.82	0.74	0.59	0.39	0.98	0.99	1	1
	(0,0,1,-1,3)	0.96	0.94	0.91	0.86	0.98	0.99	1	1
	(0.2,0.2,1,1,1)	0.8	0.72	0.59	0.38	0.98	0.99	1	1
	(1,-2,1,0.1,1)	0.83	0.76	0.64	0.46	0.99	1	1	1
100	(0,0,0,0,0)	0.75	0.64	0.46	0.24	NaN	NaN	NaN	NaN
	(0,0,0.1,0.1,0.1)	0.6	0.45	0.28	0.12	NaN	NaN	NaN	NaN
	(0,0,1,1,1)	0.94	0.89	0.8	0.68	0.98	0.99	1	1
	(0,0,1,-1,3)	0.98	0.97	0.95	0.91	0.98	0.99	0.99	1
	(0.2,0.2,1,1,1)	0.92	0.86	0.77	0.61	0.98	0.99	1	1
	(1,-2,1,0.1,1)	0.94	0.9	0.83	0.73	0.99	1	1	1
500	(0,0,0,0,0)	1	0.99	0.99	0.94	NaN	NaN	NaN	NaN
	(0,0,0.1,0.1,0.1)	0.94	0.9	0.84	0.72	NaN	NaN	NaN	NaN
	(0,0,1,1,1)	1	1	1	0.98	1	1	1	1
	(0,0,1,-1,3)	1	1	1	1	0.99	0.99	1	1
	(0.2,0.2,1,1,1)	1	1	1	0.98	1	1	1	1
	(1,-2,1,0.1,1)	1	1	1	1	1	1	1	1
1000	(0,0,0,0,0)	1	1	1	1	NaN	NaN	NaN	NaN
	(0,0,0.1,0.1,0.1)	0.98	0.97	0.94	0.88	NaN	NaN	NaN	NaN
	(0,0,1,1,1)	1	1	1	1	1	1	1	1
	(0,0,1,-1,3)	1	1	1	1	1	1	1	1
	(0.2,0.2,1,1,1)	1	1	1	1	1	1	1	1
	(1,-2,1,0.1,1)	1	1	1	1	1	1	1	1

4.3 Simulation 3: for RMST differences.

Similarly, we evaluate the Bayesian credible subgroup by using RMSTd to identify the benefiting subjects at different values of δ_{Rd} and credible level. Table 10 shows that the RMSTd that generate credible subgroups with higher credible level have higher specificity of D and lower sensitivity of D in a case of $\delta_{Rd} = 0$. Table 9 and Table 11 show that RMSTd approach has a good performance for $\delta_{Rd} \neq 0$.

Table 9: Average summary statistics for threshold $\delta_{\mathbf{RMST}} = \mathbf{-1}$ at 4 different credible levels.

Sample Size	Truth	Total Coverage				Credible Pair Size			
		40%	60%	80%	95%	40%	60%	80%	95%
50	(0,0,0,0,0)	0.96	0.98	1	1	0.28	0.42	0.6	0.8
	(0,0,0.1,0.1,0.1)	0.95	0.98	1	1	0.4	0.54	0.7	0.88
	(0,0,1,1,1)	0.71	0.82	0.92	0.98	0.47	0.58	0.69	0.82
	(0,0,1,-1,3)	0.88	0.93	0.97	0.99	0.21	0.24	0.3	0.43
	(0.2,0.2,1,1,1)	0.84	0.92	0.95	0.97	0.47	0.55	0.65	0.79
	(1,-2,1,0.1,1)	0.84	0.91	0.96	0.98	0.15	0.19	0.26	0.34
100	(0,0,0,0,0)	0.98	1	1	1	0.14	0.24	0.4	0.66
	(0,0,0.1,0.1,0.1)	0.94	0.98	0.99	1	0.22	0.34	0.52	0.74
	(0,0,1,1,1)	0.78	0.88	0.94	0.98	0.43	0.53	0.62	0.74
	(0,0,1,-1,3)	0.96	0.98	1	1	0.11	0.14	0.18	0.27
	(0.2,0.2,1,1,1)	0.91	0.97	0.99	0.99	0.34	0.42	0.52	0.68
	(1,-2,1,0.1,1)	0.9	0.96	0.98	1	0.04	0.07	0.11	0.18
500	(0,0,0,0,0)	1	1	1	1	0	0	0.01	0.04
	(0,0,0.1,0.1,0.1)	1	1	1	1	0.02	0.03	0.06	0.16
	(0,0,1,1,1)	0.82	0.9	0.96	1	0.25	0.35	0.45	0.57
	(0,0,1,-1,3)	1	1	1	1	0	0	0	0.01
	(0.2,0.2,1,1,1)	1	1	1	1	0.04	0.06	0.1	0.18
	(1,-2,1,0.1,1)	0.98	0.98	0.98	1	0.01	0.01	0.02	0.05
1000	(0,0,0,0,0)	1	1	1	1	0	0	0	0
	(0,0,0.1,0.1,0.1)	1	1	1	1	0	0.01	0.01	0.05
	(0,0,1,1,1)	0.76	0.86	0.93	0.98	0.16	0.24	0.34	0.47
	(0,0,1,-1,3)	1	1	1	1	0	0	0	0
	(0.2,0.2,1,1,1)	1	1	1	1	0	0	0.01	0.04
	(1,-2,1,0.1,1)	0.99	1	1	1	0	0	0.01	0.03

Sample Size	Truth	Sensitivity of D				Specificity of D			
		40%	60%	80%	95%	40%	60%	80%	95%
50	(0,0,0,0,0)	0.71	0.57	0.4	0.2	NaN	NaN	NaN	NaN
	(0,0,0.1,0.1,0.1)	0.59	0.46	0.3	0.12	NaN	NaN	NaN	NaN
	(0,0,1,1,1)	0.61	0.54	0.43	0.27	NaN	NaN	NaN	NaN
	(0,0,1,-1,3)	0.79	0.77	0.72	0.59	NaN	NaN	NaN	NaN
	(0.2,0.2,1,1,1)	0.55	0.48	0.38	0.23	NaN	NaN	NaN	NaN
	(1,-2,1,0.1,1)	0.91	0.9	0.88	0.85	0.99	1	1	1
100	(0,0,0,0,0)	0.86	0.76	0.6	0.34	NaN	NaN	NaN	NaN
	(0,0,0.1,0.1,0.1)	0.77	0.66	0.48	0.26	NaN	NaN	NaN	NaN
	(0,0,1,1,1)	0.71	0.65	0.59	0.43	NaN	NaN	NaN	NaN
	(0,0,1,-1,3)	0.89	0.87	0.83	0.74	NaN	NaN	NaN	NaN
	(0.2,0.2,1,1,1)	0.67	0.6	0.5	0.34	NaN	NaN	NaN	NaN
	(1,-2,1,0.1,1)	0.98	0.97	0.96	0.93	0.99	1	1	1
500	(0,0,0,0,0)	1	1	0.99	0.96	NaN	NaN	NaN	NaN
	(0,0,0.1,0.1,0.1)	0.98	0.96	0.94	0.84	NaN	NaN	NaN	NaN
	(0,0,1,1,1)	0.85	0.82	0.77	0.7	NaN	NaN	NaN	NaN
	(0,0,1,-1,3)	1	1	1	0.99	NaN	NaN	NaN	NaN
	(0.2,0.2,1,1,1)	0.96	0.94	0.9	0.82	NaN	NaN	NaN	NaN
	(1,-2,1,0.1,1)	1	1	1	1	1	1	1	1
1000	(0,0,0,0,0)	1	1	1	1	NaN	NaN	NaN	NaN
	(0,0,0.1,0.1,0.1)	1	0.99	0.99	0.95	NaN	NaN	NaN	NaN
	(0,0,1,1,1)	0.89	0.87	0.83	0.78	NaN	NaN	NaN	NaN
	(0,0,1,-1,3)	1	1	1	1	NaN	NaN	NaN	NaN
	(0.2,0.2,1,1,1)	1	1	0.99	0.96	NaN	NaN	NaN	NaN
	(1,-2,1,0.1,1)	1	1	1	1	1	1	1	1

Table 10: Average summary statistics for threshold $\delta_{\mathbf{RMST}} = \mathbf{0}$ at 4 different credible levels.

Sample Size	Truth	Total Coverage				Credible Pair Size			
		40%	60%	80%	95%	40%	60%	80%	95%
50	(0,0,0,0,0)	0.6	0.72	0.86	0.96	0.76	0.87	0.94	0.98
	(0,0,0.1,0.1,0.1)	0.67	0.8	0.89	0.97	0.75	0.86	0.93	0.98
	(0,0,1,1,1)	0.68	0.84	0.95	0.99	0.17	0.25	0.38	0.66
	(0,0,1,-1,3)	0.63	0.76	0.92	1	0.05	0.08	0.17	0.49
	(0.2,0.2,1,1,1)	0.63	0.82	0.93	0.99	0.17	0.27	0.44	0.78
	(1,-2,1,0.1,1)	0.74	0.91	0.99	1	0.2	0.34	0.59	0.76
100	(0,0,0,0,0)	0.63	0.76	0.89	0.98	0.77	0.88	0.95	0.99
	(0,0,0.1,0.1,0.1)	0.62	0.8	0.9	0.98	0.69	0.81	0.9	0.97
	(0,0,1,1,1)	0.64	0.78	0.94	0.99	0.11	0.15	0.22	0.38
	(0,0,1,-1,3)	0.63	0.79	0.9	0.99	0.03	0.05	0.08	0.21
	(0.2,0.2,1,1,1)	0.68	0.81	0.92	0.98	0.11	0.17	0.23	0.45
	(1,-2,1,0.1,1)	0.68	0.8	0.95	0.99	0.1	0.16	0.29	0.59
500	(0,0,0,0,0)	0.54	0.74	0.87	0.96	0.76	0.88	0.94	0.98
	(0,0,0.1,0.1,0.1)	0.68	0.83	0.92	0.98	0.49	0.63	0.76	0.89
	(0,0,1,1,1)	0.64	0.76	0.89	0.97	0.08	0.1	0.12	0.14
	(0,0,1,-1,3)	0.58	0.66	0.84	0.95	0.03	0.04	0.05	0.07
	(0.2,0.2,1,1,1)	0.66	0.81	0.94	0.99	0.08	0.12	0.13	0.15
	(1,-2,1,0.1,1)	0.78	0.84	0.96	0.98	0.07	0.09	0.11	0.13
1000	(0,0,0,0,0)	0.64	0.79	0.87	0.96	0.74	0.87	0.94	0.98
	(0,0,0.1,0.1,0.1)	0.64	0.78	0.92	0.98	0.35	0.46	0.76	0.77
	(0,0,1,1,1)	0.67	0.84	0.89	0.98	0.08	0.11	0.12	0.14
	(0,0,1,-1,3)	0.61	0.72	0.84	0.94	0.02	0.04	0.05	0.06
	(0.2,0.2,1,1,1)	0.62	0.74	0.94	0.97	0.07	0.1	0.13	0.14
	(1,-2,1,0.1,1)	0.74	0.84	0.96	0.98	0.06	0.08	0.11	0.13

Sample Size	Truth	Sensitivity of D				Specificity of D			
		40%	60%	80%	95%	40%	60%	80%	95%
50	(0,0,0,0,0)	NaN	NaN	NaN	NaN	0.88	0.93	0.97	0.99
	(0,0,0.1,0.1,0.1)	0.15	0.09	0.05	0.02	0.96	0.98	0.99	1
	(0,0,1,1,1)	0.74	0.65	0.5	0.25	0.97	0.98	1	1
	(0,0,1,-1,3)	0.99	0.98	0.96	0.87	0.95	0.97	0.99	1
	(0.2,0.2,1,1,1)	0.75	0.64	0.47	0.22	0.96	0.98	0.99	1
	(1,-2,1,0.1,1)	0.73	0.51	0.1	0	0.97	0.99	1	1
100	(0,0,0,0,0)	NaN	NaN	NaN	NaN	0.89	0.94	0.98	1
	(0,0,0.1,0.1,0.1)	0.18	0.11	0.05	0.02	0.95	0.97	0.99	1
	(0,0,1,1,1)	0.93	0.87	0.76	0.51	0.96	0.98	1	1
	(0,0,1,-1,3)	1	1	1	0.96	0.95	0.97	0.99	1
	(0.2,0.2,1,1,1)	0.9	0.81	0.71	0.42	0.96	0.98	0.99	1
	(1,-2,1,0.1,1)	0.94	0.87	0.62	0.08	0.97	0.98	0.99	1
500	(0,0,0,0,0)	NaN	NaN	NaN	NaN	0.87	0.93	0.97	0.99
	(0,0,0.1,0.1,0.1)	0.3	0.18	0.08	0.02	0.97	0.99	0.99	1
	(0,0,1,1,1)	1	1	1	0.98	0.95	0.97	0.99	1
	(0,0,1,-1,3)	1	1	1	1	0.95	0.96	0.98	0.99
	(0.2,0.2,1,1,1)	1	1	1	0.98	0.96	0.98	0.99	1
	(1,-2,1,0.1,1)	1	1	1	1	0.98	0.98	1	1
1000	(0,0,0,0,0)	NaN	NaN	NaN	NaN	0.89	0.94	0.97	0.99
	(0,0,0.1,0.1,0.1)	0.4	0.26	0.08	0.04	0.97	0.98	0.99	1
	(0,0,1,1,1)	1	1	1	1	0.96	0.98	0.99	1
	(0,0,1,-1,3)	1	1	1	1	0.95	0.97	0.98	0.99
	(0.2,0.2,1,1,1)	1	1	1	1	0.95	0.97	0.99	1
	(1,-2,1,0.1,1)	1	1	1	1	0.97	0.98	1	1

Table 11: Average summary statistics for threshold $\delta_{\mathbf{RMST}} = \mathbf{1}$ at 4 different credible levels.

Sample Size	Truth	Total Coverage 40%	60%	80%	95%	Credible Pair Size 40%	60%	80%	95%
50	(0,0,0,0,0)	0.96	0.98	1	1	0.3	0.43	0.63	0.82
	(0,0,0.1,0.1,0.1)	0.95	0.98	1	1	0.19	0.31	0.49	0.72
	(0,0,1,1,1)	0.72	0.82	0.92	0.98	0.13	0.19	0.25	0.31
	(0,0,1,-1,3)	0.94	0.96	0.98	1	0.03	0.05	0.08	0.13
	(0.2,0.2,1,1,1)	0.73	0.84	0.93	0.99	0.15	0.2	0.26	0.33
	(1,-2,1,0.1,1)	0.99	1	1	1	0	0	0	0.02
100	(0,0,0,0,0)	1	1	1	1	0.14	0.23	0.39	0.66
	(0,0,0.1,0.1,0.1)	0.96	0.99	1	1	0.09	0.16	0.27	0.5
	(0,0,1,1,1)	0.87	0.93	0.98	0.98	0.09	0.13	0.19	0.25
	(0,0,1,-1,3)	0.98	1	1	1	0.01	0.01	0.03	0.05
	(0.2,0.2,1,1,1)	0.86	0.95	0.98	1	0.1	0.15	0.19	0.26
	(1,-2,1,0.1,1)	1	1	1	1	0	0	0	0
500	(0,0,0,0,0)	1	1	1	1	0	0	0.01	0.05
	(0,0,0.1,0.1,0.1)	1	1	1	1	0	0.01	0.02	0.06
	(0,0,1,1,1)	0.97	0.99	0.99	1	0.02	0.03	0.05	0.1
	(0,0,1,-1,3)	1	1	1	1	0	0	0	0
	(0.2,0.2,1,1,1)	1	1	1	1	0.01	0.02	0.04	0.08
	(1,-2,1,0.1,1)	1	1	1	1	0	0	0	0
1000	(0,0,0,0,0)	1	1	1	1	0	0	0	0
	(0,0,0.1,0.1,0.1)	1	1	1	1	0	0	0	0.01
	(0,0,1,1,1)	1	1	1	1	0	0	0.02	0.05
	(0,0,1,-1,3)	1	1	1	1	0	0	0	0
	(0.2,0.2,1,1,1)	1	1	1	1	0	0	0.01	0.02
	(1,-2,1,0.1,1)	1	1	1	1	0	0	0	0

Sample Size	Truth	Sensitivity of D 40%	60%	80%	95%	Specificity of D 40%	60%	80%	95%
50	(0,0,0,0,0)	NaN	NaN	NaN	NaN	0.99	1	1	1
	(0,0,0.1,0.1,0.1)	NaN	NaN	NaN	NaN	1	1	1	1
	(0,0,1,1,1)	0.4	0.3	0.19	0.1	1	1	1	1
	(0,0,1,-1,3)	0.95	0.92	0.89	0.81	1	1	1	1
	(0.2,0.2,1,1,1)	0.4	0.27	0.17	0.08	0.99	1	1	1
	(1,-2,1,0.1,1)	NaN	NaN	NaN	NaN	1	1	1	1
100	(0,0,0,0,0)	NaN	NaN	NaN	NaN	1	1	1	1
	(0,0,0.1,0.1,0.1)	NaN	NaN	NaN	NaN	1	1	1	1
	(0,0,1,1,1)	0.56	0.46	0.33	0.2	1	1	1	1
	(0,0,1,-1,3)	0.98	0.98	0.96	0.91	1	1	1	1
	(0.2,0.2,1,1,1)	0.56	0.42	0.33	0.2	1	1	1	1
	(1,-2,1,0.1,1)	NaN	NaN	NaN	NaN	1	1	1	1
500	(0,0,0,0,0)	NaN	NaN	NaN	NaN	1	1	1	1
	(0,0,0.1,0.1,0.1)	NaN	NaN	NaN	NaN	1	1	1	1
	(0,0,1,1,1)	0.9	0.85	0.74	0.56	1	1	1	1
	(0,0,1,-1,3)	1	1	1	1	1	1	1	1
	(0.2,0.2,1,1,1)	0.95	0.92	0.83	0.66	1	1	1	1
	(1,-2,1,0.1,1)	NaN	NaN	NaN	NaN	1	1	1	1
1000	(0,0,0,0,0)	NaN	NaN	NaN	NaN	1	1	1	1
	(0,0,0.1,0.1,0.1)	NaN	NaN	NaN	NaN	1	1	1	1
	(0,0,1,1,1)	0.99	0.98	0.91	0.77	1	1	1	1
	(0,0,1,-1,3)	1	1	1	1	1	1	1	1
	(0.2,0.2,1,1,1)	1	0.99	0.97	0.89	1	1	1	1
	(1,-2,1,0.1,1)	NaN	NaN	NaN	NaN	1	1	1	1

5 Model diagnosis

5.1 The prostate cancer dataset

From the posterior density of the model parameters in Fig 1, it shows that the distributions are approximately normal.

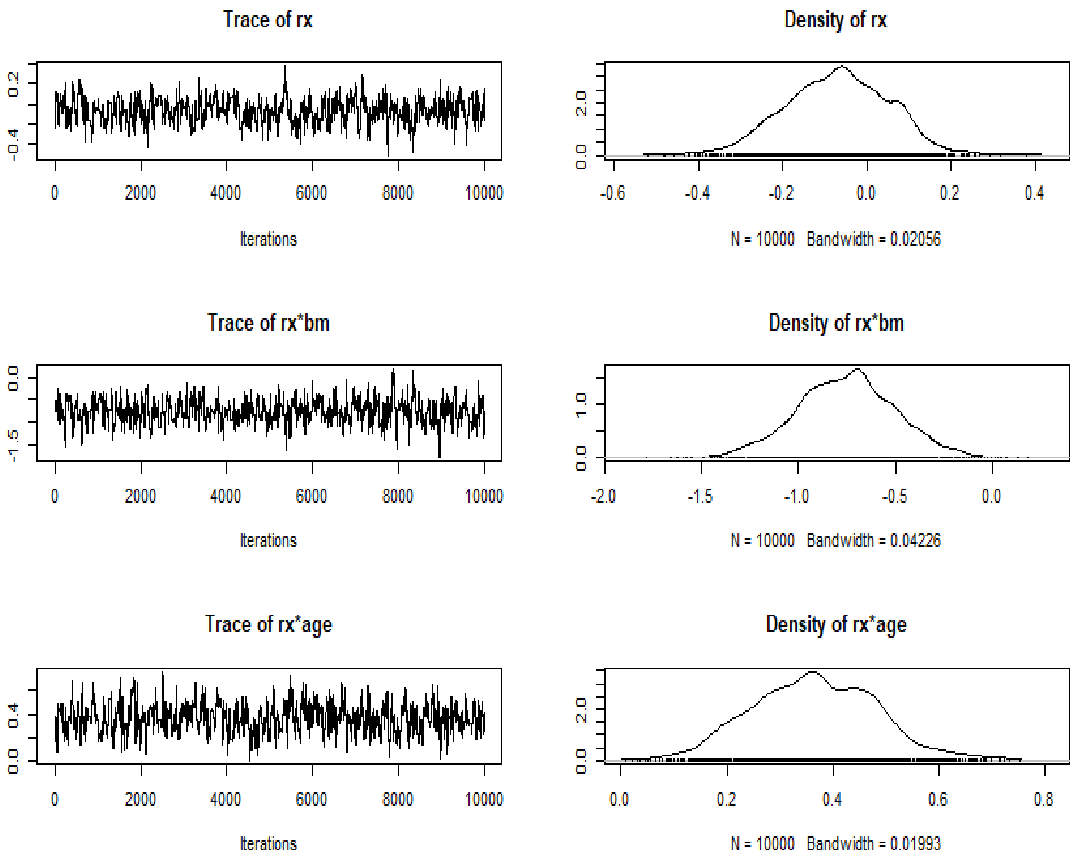

Figure 1: Left panel is the trace plot. Right panel is the posterior density of the model parameters based on 10000 draws kept after 500 burn–in iterations from the joint posterior sample.

5.2 A large simulated clinical trial dataset

Similarly, we show the posterior density of the model parameters in Fig 2, and it shows that the distributions are approximately normal.

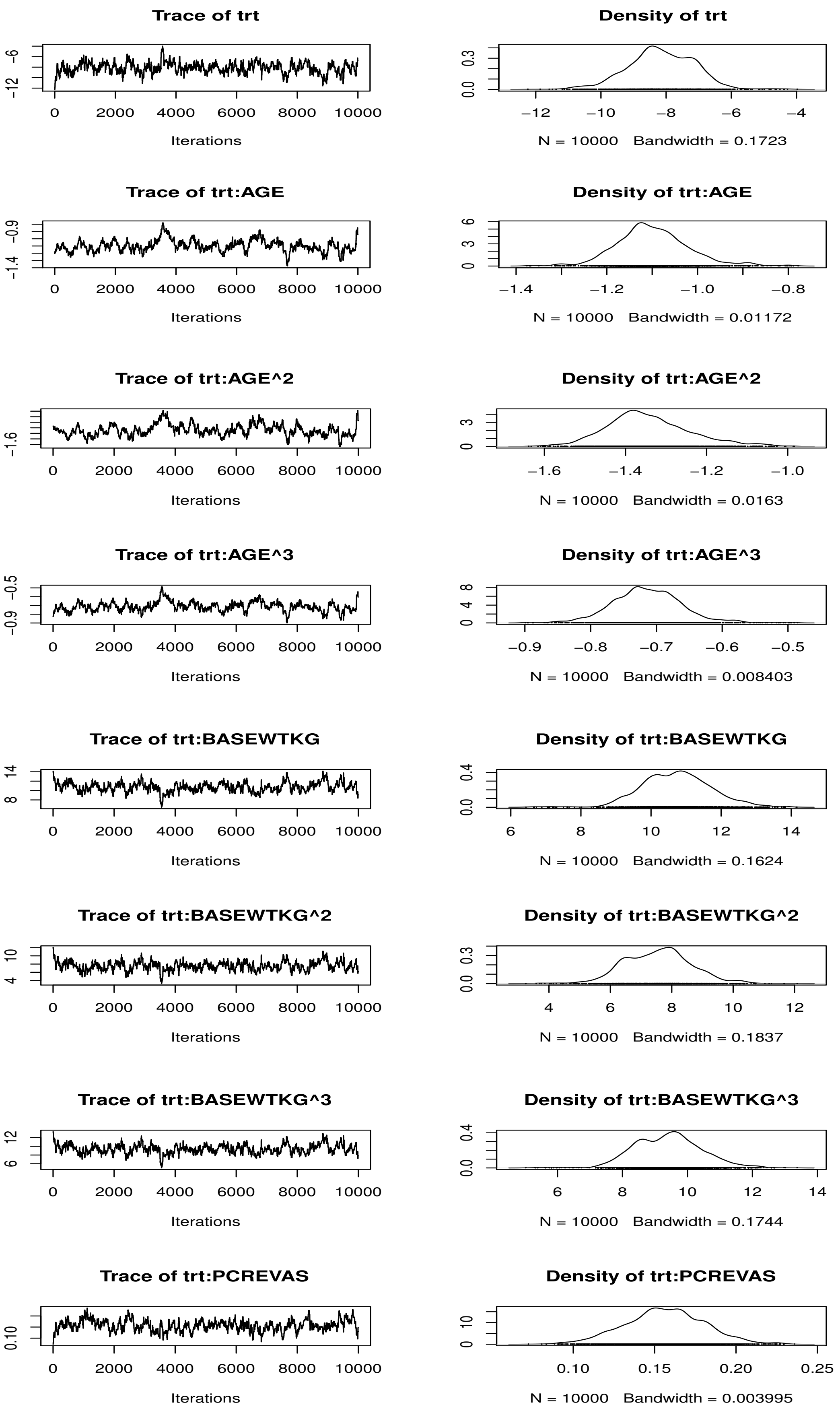

Figure 2: Left panel is the trace plot. Right panel is the posterior density of the model parameters based on 10000 draws kept after 500 burn–in iterations from the joint posterior sample.

6 Pointwise method

We demonstrate the pointwise method as a benchmark method to compare with our proposed method. The pointwise method uses the same Cox regression model as our method , but it does not account for multiplicity in constructing credible subgroups. Precisely, the exclusive credible subgroup D contains the covariate points at which the posterior probability of $\Delta(z) > \delta$ is greater than $1 - \alpha$. The inclusive credible subgroup S includes the covariate points at which the posterior probability of $\Delta(z) \leq \delta$ is at most α.

6.1 Simulation study

Table 12 represents the Bayesian credible subgroup by using pointwise method. It shows that log HR and RMST yield similar result, and the total coverage is mostly smaller than 80%. Moving from pointwise method to our proposed methods, there is increasing in credible pair size, specificity of D, but smaller sensitivity of D.

Table 12: Pointwise method. Average summary statistics for **80%** credible level.

Sample Size	Truth	Total Coverage		Credible Pair Size		Sensitivity of D		Specificity of D		MSE	
		log HR	RMST	log HR	RMST	log HR	RMST	log HR	RMST	log HR	RMST
50	(0,0,0,0,0)	0.55	0.4	0.7	0.58	NaN	NaN	0.87	0.8	0.44	0.48
	(0,0,0.1,0.1,0.1)	0.51	0.35	0.65	0.54	0.2	0.28	0.92	0.88	0.46	0.51
	(0,0,1,1,1)	0.7	0.63	0.2	0.17	0.65	0.71	0.97	0.97	0.46	0.38
	(0,0,1,-1,3)	0.8	0.74	0.08	0.07	0.99	0.99	0.98	0.97	0.96	0.28
	(0.2,0.2,1,1,1)	0.73	0.65	0.19	0.16	0.69	0.75	0.97	0.96	0.43	0.36
	(1,-2,1,0.1,1)	0.79	0.73	0.19	0.19	0.76	0.74	0.98	0.97	0.59	0.26
100	(0,0,0,0,0)	0.5	0.4	0.69	0.57	NaN	NaN	0.85	0.79	0.2	0.27
	(0,0,0.1,0.1,0.1)	0.54	0.35	0.65	0.53	0.25	0.32	0.91	0.88	0.18	0.26
	(0,0,1,1,1)	0.76	0.68	0.14	0.12	0.83	0.87	0.98	0.97	0.23	0.24
	(0,0,1,-1,3)	0.79	0.73	0.06	0.05	1	1	0.97	0.97	0.54	0.14
	(0.2,0.2,1,1,1)	0.74	0.6	0.15	0.11	0.84	0.9	0.97	0.96	0.22	0.2
	(1,-2,1,0.1,1)	0.82	0.73	0.12	0.1	0.92	0.93	0.98	0.97	0.28	0.19
500	(0,0,0,0,0)	0.48	0.38	0.72	0.62	NaN	NaN	0.85	0.8	0.03	0.05
	(0,0,0.1,0.1,0.1)	0.64	0.55	0.47	0.38	0.3	0.38	0.97	0.95	0.03	0.05
	(0,0,1,1,1)	0.74	0.65	0.1	0.09	1	1	0.97	0.96	0.08	0.06
	(0,0,1,-1,3)	0.86	0.79	0.05	0.04	1	1	0.98	0.97	0.31	0.05
	(0.2,0.2,1,1,1)	0.79	0.69	0.11	0.09	1	1	0.98	0.97	0.08	0.06
	(1,-2,1,0.1,1)	0.8	0.72	0.09	0.07	1	1	0.98	0.97	0.07	0.07
1000	(0,0,0,0,0)	0.52	0.4	0.71	0.6	NaN	NaN	0.86	0.8	0.02	0.03
	(0,0,0.1,0.1,0.1)	0.68	0.56	0.36	0.28	0.38	0.47	0.97	0.96	0.02	0.03
	(0,0,1,1,1)	0.76	0.68	0.11	0.09	1	1	0.98	0.96	0.06	0.03
	(0,0,1,-1,3)	0.86	0.79	0.05	0.04	1	1	0.98	0.97	0.28	0.03
	(0.2,0.2,1,1,1)	0.72	0.65	0.1	0.08	1	1	0.97	0.96	0.07	0.04
	(1,-2,1,0.1,1)	0.86	0.75	0.09	0.08	1	0.98	0.99	0.98	0.05	0.05

6.2 Prostate cancer dataset

The left panel in Fig 3 shows credible subgroups, for prostate cancer patients, using log HR and credible level of 95%. We used the same value $\delta_H = 1$ to define subgroups as in our manuscript. Similarly, the right panel shows credible subgroups using the RMSTd with a credible level of 95% and $\delta_R = 0$. For patients with or without existence of bone metastasis, the pointwise method provides tighter uncertainty region in both Δ_H and Δ_{Rd} than our proposed method does. As a result, the exclusive credible subgroup D from pointwise method is larger than our proposed method. In addition, the difference in RMSTs provides a larger exclusive subgroup D than a log HR method does.

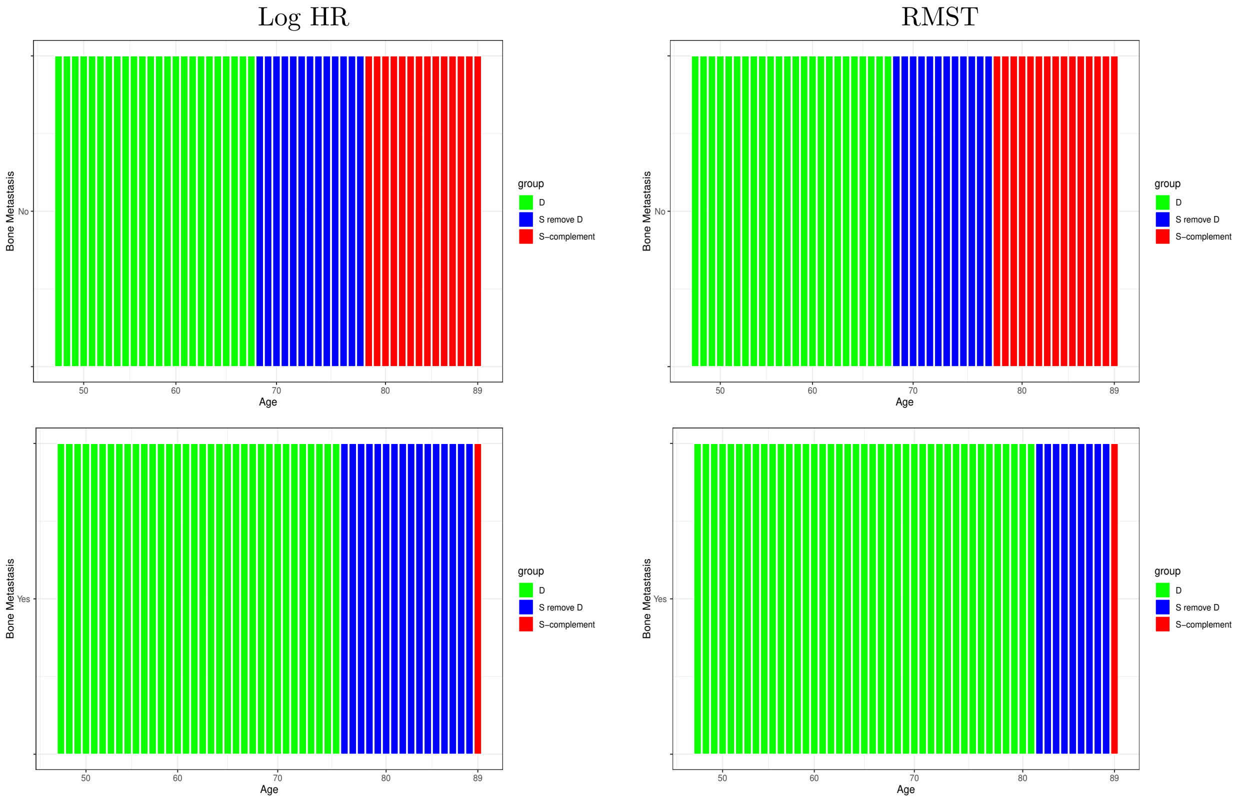

Figure 3: Poitwise method in constructing credible subgroups for prostate cancer dataset.

6.3 A large simulated clinical trial dataset

Fig 4 and Fig 5 shows credible subgroups using the log HR and difference in RMST, respectively. We used the same value settings to define subgroups as in our manuscript. The results show that the pointwise method provides a tighter uncertainty region in both Δ_H and Δ_{Rd} than our proposed method does. As a result, the exclusive credible subgroup D from pointwise method is larger than our proposed method.

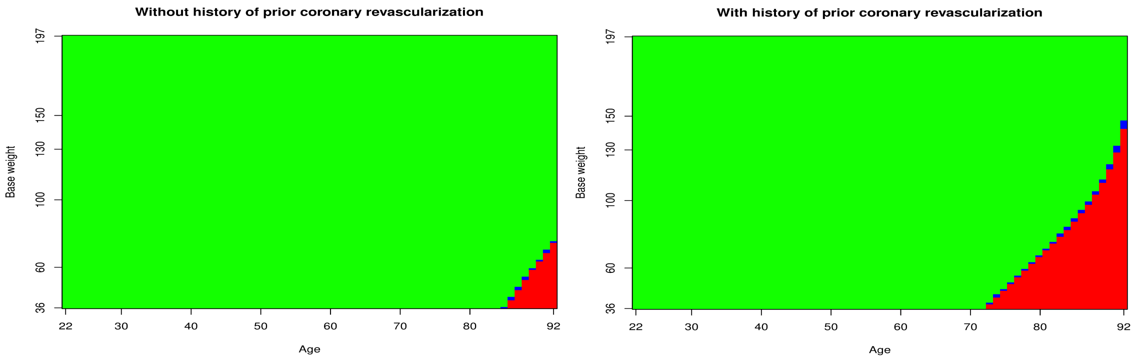

Figure 4: Log HR as PTE. Poitwise method in constructing credible subgroups for prostate cancer dataset.

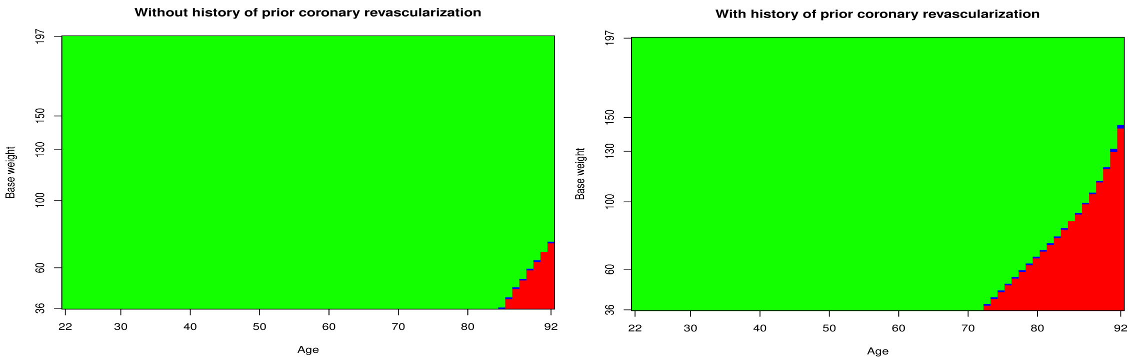

Figure 5: Difference in RMST as PTE. Poitwise method in constructing credible subgroups for prostate cancer dataset.

7 Simulation study under nonproportional hazard assumption

Table 13–15 diagnosis results for RMSTd under nonproportional hazard assumption. When the sample size is increasing, the total coverage, sensitivity and specificity of D increase, but credible pair size and MSE decrease. Moreover, the more conservative coverage has lower sensitivity of D but higher specificity of D.

Table 13: Average summary statistics for 40% credible subgroup pairs under nonproportional hazard assumption

Sample Size	Total Coverage	Credible Pair Size	Sensitivity of D	Specificity of D	MSE
50	0.36	0.18	0.83	0.36	0.68
100	0.4	0.14	0.88	0.4	0.58
500	0.44	0.06	0.97	0.44	0.43
1,000	0.54	0.06	0.98	0.54	0.38

Table 14: Average summary statistics for 60% credible subgroup pairs under nonproportional hazard assumption

Sample Size	Total Coverage	Credible Pair Size	Sensitivity of D	Specificity of D	MSE
50	0.56	0.31	0.71	0.56	0.68
100	0.56	0.22	0.81	0.56	0.58
500	0.62	0.1	0.94	0.62	0.43
1,000	0.72	0.09	0.96	0.72	0.38

Table 15: Average summary statistics for 95% credible subgroup pairs under nonproportional hazard assumption

Sample Size	Total Coverage	Credible Pair Size	Sensitivity of D	Specificity of D	MSE
50	0.95	0.75	0.26	0.95	0.68
100	0.98	0.6	0.43	0.98	0.58
500	0.98	0.3	0.75	0.98	0.43
1,000	1	0.22	0.84	1	0.38
